# Pathophysiology of chronic rhinosinusitis, pharmaceutical therapy options

**DOI:** 10.3205/cto000124

**Published:** 2015-12-22

**Authors:** Claus Bachert, Gabriële Holtappels

**Affiliations:** 1 Department of Otolaryngology and Upper Airways Research Laboratory, University of Ghent, Belgium; 2 Division of ENT Diseases, CLINTEC, Karolinska Institute, University of Stockholm, Sweden

**Keywords:** chronic rhinosinusitis, pathophysiology, cluster analysis, phenotypes, endotypes, Staphylococcus aureus, biologics

## Abstract

Research in immunology has brought great progress in knowledge of inflammatory processes in the last 2 decades, which also has an impact on the upper airways. Our understanding of the pathophysiology of chronic rhinosinusitis developed from a rather mechanistic point of view with a focus on narrow clefts and mucociliary clearance to the appreciation of a complex network of immunological pathways forming the basis of disease. We today differentiate various forms of inflammation, we start to understand complex immune-regulatory networks and the reasons for their failure, and have already developed innovative approaches for therapy for the most severely ill subjects. Due to this new knowledge in inflammation and remodeling processes within mucosal tissue, specifically on the key driving factors, new diagnostic tools and therapeutic approaches for chronic rhinosinusitis have developed; the differentiation of endotypes based on pathophysiological principles will be crucial for the use of innovative therapies, mostly humanized monoclonal antibodies. Several hundred of those antibodies are currently developed for various indications and will impact our specialty as well as pneumology to a great extent.

## 1 Introduction: From pathophysiology to endotypes of chronic rhinosinusitis and their treatment

Chronic rhinosinusitis (CRS) describes a heterogeneous group of diseases of the nose and the paranasal sinuses that is characterized by two phenomena: inflammation and tissue remodeling. According to the European position paper on rhinosinusitis and nasal polyps [1], CRS is defined by the presence of at least two of the following symptoms: nasal obstruction, nasal secretion and/or post-nasal drip (PND), headaches and/or facial pains, a reduction of smelling for more than 12 weeks during the last year while at least one of the first two mentioned symptoms should be observed. The burden of the symptoms, the associated reduction of the quality of life and the influence of the disease on work productivity are often underestimated. With reference to the endoscopic findings, the difference is made between the clinical phenotypes of CRS without and CRS with nasal polyps (CRSsNP, CRSwNP) [1]. Recent findings on remodeling confirm this differentiation and reveal that there are significant differences between the phenotypes. Histologically, CRSsNP is characterized by fibrosis of the mucosa and the basal membrane while nasal polyposis is characterized by important edema with deposition of albumin and the development of pseudocysts. The differentiation of the inflammation types based on T helper cells allows a more differentiated classification according to pathomechanical principles into so-called endotypes within the clinical phenotypes which can finally be used to define innovative therapeutic objectives and to implement them in daily clinical routine.

CRS is one of the most frequently occurring diseases in humans. Recently, the Global Allergy and Asthma Network of Excellence GALEN [2] published a first multicenter prevalence study on chronic rhinosinusitis that is based on 56,000 questionnaires that were returned by 19 centers in 12 European countries; a random sample was also submitted to clinical examination by a specialist in order to confirm the diagnosis [3]. The investigation came to the conclusion that the prevalence of CRS in Europe amounts to 10.9%, between 6.9 and 27.1% in different European cities [4]. Higher prevalences were discovered in younger people, women, and southern countries; smoking significantly increased the risk for CRS, depending on the dose. In the USA the prevalence is even higher [5]. CRS, however, also affects and increased part of the older population up to the age of 60, afterwards the incidence decreases. Among all CRS patients in the USA, about 20–33% suffer from CRSwNP, 60–65% suffer from CRSsNP, and 8–12% from allergic fungal sinusitis (AFS).

One important finding of the GALEN study on chronic rhinosinusitis was that CRS is associated with an increased risk for a special type of asthma, i.e. a so-called late-onset asthma or asthma occurring after the age of 18 [6]. This association was independent from an existing allergy, in contrast to asthma in early childhood which is clearly associated with allergic rhinitis. The exact pathophysiology of this observation is not known up to now. Mechanisms could consist of the nasobronchial reflex, aspiration of nasal secretion, inhalation of cold air with obstructed nose, or the progress of the inflammation along the airway mucosa. The research program established in the USA entitled “Severe Asthma Research Program (SARP)” recently identified five different asthma phenotypes in a cluster analysis; in one of the clusters with non-atopic late-onset asthma, more than half of the patients reported about interventions of the paranasal sinuses [7]. Another investigation of 2,500 asthmatics revealed that about 51% had rhinosinusitis; CRS was associated with a deterioration of coughing symptoms and with a higher number of exacerbations [8]. Those results confirm the relationship between CRS and asthma; however, it remains unclear for which type of CRS they are true and which pathomechanisms are responsible.

Further it is well-known that part of the patients with CRS will have recurrences even after well performed surgery and will have to undergo another surgical intervention – or innovative therapeutic approaches (see the following chapters) [1]. Own observations show that nearly 80% of CRSwNP patients develop at least one recurrence over a time of 12 years after complete surgery of all paranasal sinuses and removal of all polyps. About 50% of those patients have to undergo at least one further surgery (data not published). It would be desirable to understand the pathomechanisms of this disposition to develop recurrences and to recognize patients with a high disposition in the group of CRSwNP patients already at the time of first surgery [9] in order to be able to adapt treatment strategies.

It is not possible to fulfill this task only with endoscopes and CT scans; a basic knowledge of the pathomechanisms of inflammation and of remodeling is required as well as differentiation of CRS into relevant endotypes based on this knowledge. In the future biomarkers will be applied for their identification. The understanding of the pathomechanisms will also define new therapeutic objectives that will open new possibilities for the management of especially severe types of airway diseases. Those endotypes will allow to give statements on the prognosis of the disease and their comorbidities and to apply specific innovative biologics, generally humanized monoclonal antibodies for cytokines or their receptors, in those patients who have the highest therapeutic potential.

The present paper will first discuss the significant aspects and regulators of inflammation and remodeling that are then attributed to the different endotypes on which finally innovative therapeutic approaches are based. In this context, very special pathomechanisms and relationships are explained that play a role in the development of endotypes and that help understanding the diseases and their secondary consequences. Also the innovative therapeutic approaches will be understandable and clear. However, those explanations make no claim to be complete.

## 2 Selected pathomechanical principles of the upper airways

The mucosa of the upper airways represents a barrier of the body between the environment with pollutants, viruses, and bacteria and the body’s defense system that can be classified into an unspecific “innate” and a specific “adaptive” defense. Overlapping of these systems is profitable. Starting with genetics via the innate and adaptive defenses up to the mechanisms that act against inflammation in healthy people, deficits were found in CRS patients that in total cause or at least allow an affection of the mucosa. In the environment, those defects have to face invading substances, viruses, and bacteria that complete and potentiate each other and that, as for example in the case of *Staphylococcus aureus* and *Pseudomonas aeruginosa*, may establish in the mucosa. This mechanism leads to a severe inflammatory reaction that in case of *Staph. aureus* is further intensified by the immune proteome of the germ.

### 2.1 Genetic and epigenetic findings in chronic rhinosinusitis

Single nucleotide polymorphism (SNP) is a variation of the DNA sequence where the genome of individuals of a biologic species is different in the position of one single nucleotide – A, T, C, or G. Such genetic variations are for example responsible for predispositions of diseases and the body’s reaction on environmental stimuli. Up to now, studies on CRS identified 53 single nucleotide polymorphisms (SNP) that are associated with the phenotypes of CRSwNP or CRSsNP. However, only a pooled genome-wide association study (pGWAS) was performed [10]. Most association studies (see Table 1(Tab. 1)) examined only specific genes of which the products contribute to the innate immune defense or inflammatory reactions because findings in those genes are probable in the context of inflammatory diseases. Unfortunately, many of those studies including the Canadian pGWAS (173 patients and 130 controls with CRS) were based on patient populations of relatively small size so that the knowledge on the genetics of CRS is rather limited [10], [11].

Recently we investigated on the reproducibility of all SNP associations with CRSsNP and CRSwNP described up to now in a group of Caucasians of European origin [12]. According to the current guidelines, CRS had been diagnosed based on nasal endoscopy and computed tomography. The study population consisted of 275 patients with CRSwNP and 338 patients with CRSsNP as well as a series of controls from a publicly available database. This study provided only 7 SNPs that could be reproduced and that are thus most probably also relevant for our patients; however, the existence of further relevant associations cannot be excluded.

Regarding the SNP Rs2873551 in the gene of prolyl tRNA synthetase 2 (PARS2), there was a strong and significant relationship with CRS; this SNP had already been identified in the Canadian pGWAS. PARS2 activates amino acids for protein synthesis by producing aminoacyladenylates. Inhibition of the function of PARS2 causes suppression of the cellular growth and could have an impact on the cellular proliferation in combination with inflammatory processes and the innate immune defense. The SNP rs1800469 in the gene of TGF-β1 has been associated with chronic obstructive pulmonary disease and rhinosinusitis in asthma patients [13]; this cytokine will be discussed later in the context of remodeling of CRS. Also SNP rs1483757 in the nitric oxide synthase 1 gene and the SNP rs4657164 in the nitric oxide synthase 1 adapter protein gene are associated with CRS and are also found in genes that play a role in asthma and allergic rhinitis [14]. Nitric oxide also plays an important role in the pathophysiology of asthma [15] and in the defense against specific bacteria including *Pseudomonas aeruginosa* [16].

Comparing CRSwNP and CRSsNP patients, further associations could be found. The SNP rs4504543 in the gene of acyloxyacylhodrolase (AOAH) could lead to a disturbed degradation of lipopolysaccharides [17]. In the pGWAS, this SNP had already been related to the CRS phenotype [10] which was confirmed by a Chinese patient population [17]. Furthermore, an association with asthma was identified [18].

So it is obvious that the genetic variations that were found in our investigation had already been associated with CRS and partly also with asthma or allergic rhinitis and thus could play a role in diseases of the airways. The detected associations, however, are independent from the aspect if patients suffer from asthma or allergic rhinitis. The SNPs in the gene of PARS2, in the gene of TGF-β1, and in the NOS1 gene are suitable candidates for further analyses because also from a biological point of view they are plausible.

Also in the context of epigenetic processes that significantly influence the development and manifestation of the pattern of the inflammation, tissue specific chronic inflammatory processes are subject to modifications. Epigenetic mechanisms are classified into DNA methylation, histone acetylation, and the formation of non-coding RNA, so-called miRNA, that influences the expression of other genes. This is another level of regulation between genes and proteins that can decisively be modified by inflammatory processes and also by the environment.

Among others, epigenetic processes lead to functional modifications in the genome without change or modification of the basic DNA nucleotide sequence. Examples of such modifications are DNA methylation and histone modifications that both lead to a reduction of the expression of a gene; however, this may result in an amplification or attenuation of the inflammation, depending on the genes that are expressed in a reduced way. A first investigation on methylation modifications in CRSwNP found DNA hypermethylation in 332 loci of 296 genes. The relevant gene products contributed to lymphocyte and cellular proliferation, to the activation of leukocytes, to the biosynthesis and secretion of cytokines, to immune reactions, to inflammatory processes, and to the immunoglobulin synthesis [19]. In comparison, hypomethylation was identified in 158 loci of 141 genes with involvement in ectodermal development, in hemostasis, in wound healing, in the binding of calcium ions, and in oxidoreductase activity. It is of high significance that within the arachidonic acid metabolism loci were hypomethylated in genes responsible for prostaglandin-D-synthase (PDGS), for 5-lipoxidenase activating protein (ALOX5AP), and for leukotriene-B4 receptor (LTB4R), while hypermethylation was found in the gene for prostaglandin-E-synthase (PGES) [19]. DNA methylation processes can be initiated by the release of toxic messenger substances because of the activation of eosinophils [20] that may lead to gene silencing by expressing hypohalogenous acids; they are the consequence of eosinophilic diseases, histone hyperacetylations can lead to an inhibition of the TGF-β1 induced Smad 2/3, to the restoring of Smad7 signal suppressed by TGF-β1, and to a blockage of the proliferation of TGF-β1 induced nasal polyp fibroblasts [21], [22].

The genetic transcription is also regulated by non-coding RNA, e.g. by micro RNA and by long, non-coding miRNA. An increased expression of miR-125b in the epithelium of eosinophilic CRSwNP patients in comparison to controls and non-eosinophilic CRS patients, for example, aims at 4E-BP1, a translation repressor, suppressing type-1 interferon genes [23]. Those genes are needed for the defense against viral infections. This defense can be disturbed in those patients (actually a suppression of IFN production against herpes simplex viruses (HSV) and rhinoviruses in patients with eosinophilic nasal polyposis is found, in contrast to control groups, see 2.5.1). 

In an own investigation we assessed the regulation of an inflammation in two well-defined endotypes, interleukin(IL)-5-positive and IL-5-negative CRSwNP, by miRNAs in a genome-wide approach by means of miRCURY LNA microarray platform in mucosal tissue. A total of 86 miRNAs were differentially expressed and could be classified into 5 different profiles; the investigation indicates regulatory mechanisms in the mucous production, the collagen synthesis, the cytochromes P450, and the glycosphingo-lipid-biosynthesis that is regulated via miRNAs (Zhou Peng, in press). Hence, there are new possibilities to examine further pathomechanisms, and probably therapeutic approaches in the future, because miRNAs can also be blocked.

### 2.2 Congenital immune defense: mechanisms and deficits

The upper airways are covered by a mucosal layer consisting of ciliated, pseudo-stratified columnar epithelial cells (EC) and mucus producing goblet cells. The drainage of the mucus is achieved by active mucociliary activity, not by gravitation. The mucosa is covered by mucus where micro-organisms, dust, and stimulating particles get caught. Nasal secretion consists of epithelial cells, goblet cells, epithelial cell proteins, lacrimal fluid, and vascular transudation. The primary protein parts of this secretion are mucin glycoproteins with their peptide structure and the oligosaccharide side chains. Those glycoproteins decisively influence the consistency of the mucus and furthermore they influence the interaction between host and microorganisms [24]. Mucins bind surface adhesins on microorganisms and thus inhibit their capacity to colonize the epithelium. Mucociliary activity transports the mucus from the paranasal sinuses into the nasal cavity and the pharynx where it is swallowed. The relevance of this process for the health of the airways becomes obvious in the high prevalence of sinusitis in patients with genetic defects influencing the mucociliary flow, as for example in cases of cystic fibrosis (chloride transport) or Kartagener syndrome (dyskinesia of the cilia). The nose has a large mucosal surface with a mucus layer that filters air particles and tempers and moistens the air flowing over it. In the nasal submucosa, vascular plexus are found that swell after exposition to certain triggers such as stimuli, temperature changes etc.; further more mucus is produced.

Mucin 5AC and mucin 5B are the most important secreted mucins and determine the consistency of the mucus significantly. Both are especially up-regulated in eosinophilic CRSwNP. Recently we could show that the regulation of both mucins occurs via the Th2 cytokines IL-4 and IL-13 while IL-5 has no influence [25]. 

#### 2.2.1 Antimicrobial peptides

Epithelial cells release a wide range of antimicrobial molecules, as for example enzymes (lysozyme, chitinases, and peroxidases), opsonins (complement and pentraxin-3), permeabilizing proteins (defensins, cathelicidins), C1 complexes (surfactant protein A, surfactant protein D, and mannose-binding lectin), and ligands (lactoferrin and mucins) [26], [27]. Beside their antimicrobial effect they have different other effects on cellular differentiation and wound healing [28]. The protein PLUNC (palate, lung, and nasal clone protein) is secreted by glandular epithelium and not by surface epithelium. Probably it is of particular importance for airway diseases because it has biofilm inhibiting properties.

A weak host defense could be the result of a primary defect of the innate immunity in the paranasal sinuses, but it is also possible that it is a consequence of chronic eosinophilic airway inflammation. There are hints that Th2 cytokines might down-regulate the production of mediators of the innate immunity, such as for example human beta defensing 2 and surfactant protein A [29]. Other cytokines, in particular T cell cytokine IL-22, induce mediators of the innate immune defense in epithelial cells by activating STAT3 (transcription factor signal transducer and activator of transcription 3) which is a mediator of mucosal host defense and epithelial repair mechanisms [30], [31]. Th2 polarization may lead to a reduced expression of IL22 receptor and STAT3 signal pathway [32], [33].

Also taste receptors (taste receptor family 2 (T2R)), also called “bitter taste receptors”, that were first described in the tongue, are expressed in the airways and assess toxic chemicals but also signal of bacteria they release for communication among each other. In this way, the receptor T2R38 is activated by secreted products of *Pseudomonas aeruginosa* which leads to the induction of nitric oxide and finally to bacterial death [34]. Genetic defects of the receptor increase the risk of infection with gram-negative germs.

#### 2.2.2 Epithelial barriers

Respiratory epithelial cells are connected via adhesion complexes, consisting of apical tight junctions, intermediate junctions, desmosomes, and hemi-desmosomes. A reduced expression of the tight junction proteins of claudin and occludin [35] or desmosomal proteins [36] can play a key role in the etiology of diseases of the upper airways. Reduced concentrations of protease inhibitors, such as the serine protease inhibitor Kazal type 5 (LEKT1), in the epithelium may lead to an increased sensitivity for the activity of endogenous and exogenous proteases [37]; fungi, bacteria, and many allergens dispose of a significant intrinsic protease activity. A simultaneous weakness of endogenous protease inhibitors such as LEKT1 could have the effect that the mechanical barrier cannot counter a protease attack so that foreign proteins can pass the mucosal barrier more easily.

**Figure 1:**
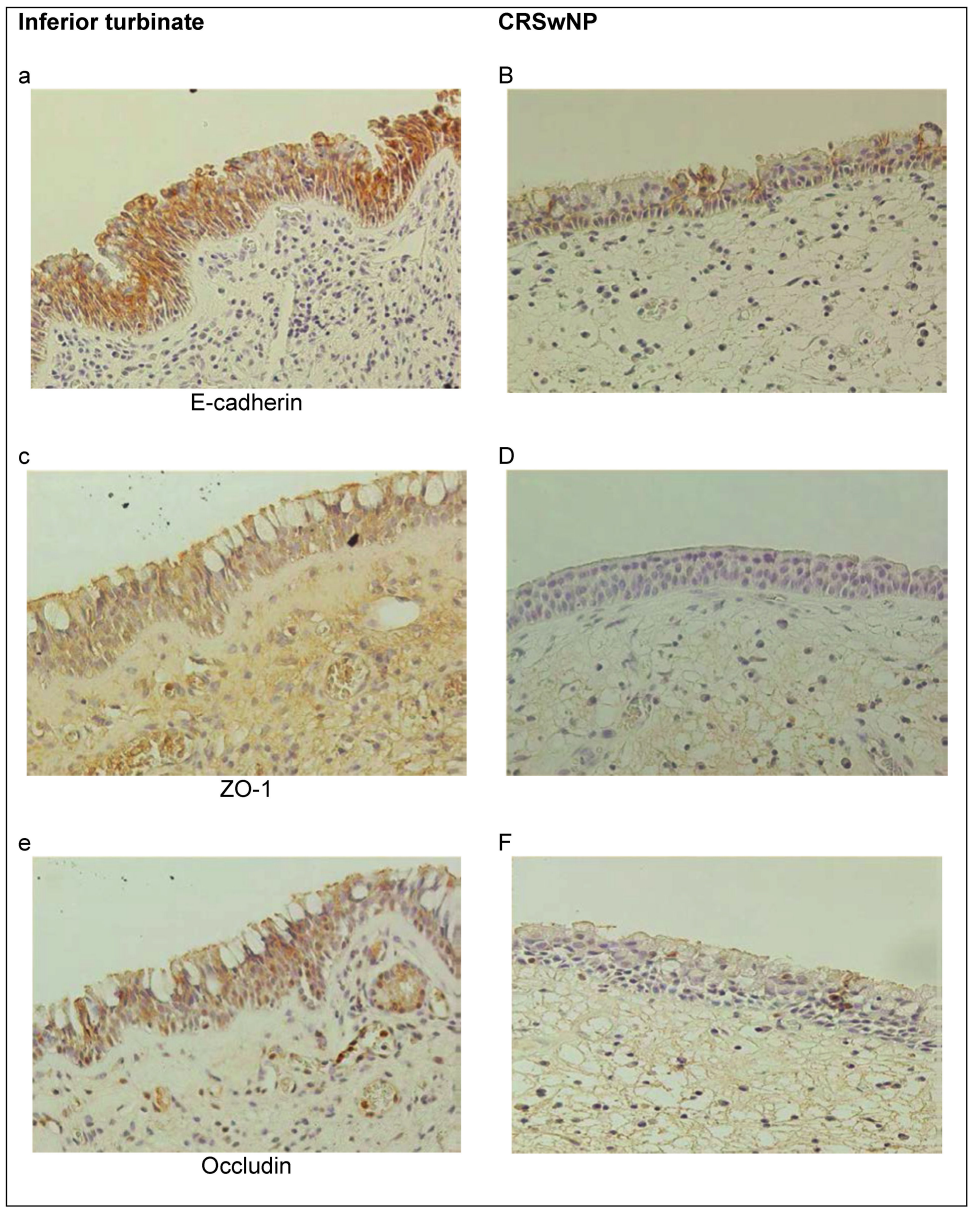
Expression of tight junction molecules in patients with CRSwNP in comparison to healthy people (inferior turbinate): significantly reduced expression leading to weak epithelial barrier in CRSwNP

In nasal polyps, occludin, the Zonula occludens 1 protein, and E-cadherin are expressed in a reduced way so that the epithelial barrier can be passed more easily by viral and bacterial infections [38], [39] (Figure 1 (Fig. 1)).

#### 2.2.3 Receptors of congenital immune defense: Toll-like, NOD-like, and RIG-like receptors

Epithelial cells of the nose and the paranasal sinuses do not only represent a physical barrier, but have active functions in the context of innate and acquired immune defense [40]. They express membrane-bound and cytoplasmatic pattern recognition receptors (PRR) that recognize pathogen-associated molecular patterns (PAMPs) [41]. The activation of those receptors leads to the release of chemokines and cytokines and components of the innate immune system activating further immune cells (for example neutrophils). Different microbial factors or PAMPs, such as for example bacterial lipopolysaccharides (LPS), flagellin, lipoteichoic acid, peptidoglycan, double stranded RNA, and unmethylized CpG motives, can be recognized by many PRRs [42]. The activation of PRRs in the nasal epithelium may lead either to the induction of tolerance or to the expression of inflammatory mediators attracting neutrophil lymphocytes.

Several groups of such receptors were described recognizing PAMPs, among them toll-like receptors (TLR), nucleotide-binding oligomerisation domain (NOD) like receptors, retinoic acid induced gene (RIG)-1-like receptors, and the melanoma differentiation associated gene (MDA)-5. The complex of TLR currently encompasses 10 integral membrane glycoproteins that recognize extracellular and intracellular PAMPs [43]. All TLR signaling pathways lead to activation of the transcription factor NF-κB (nuclear factor kappa B) which controls the expression of numerous genes for inflammatory cytokines. Obviously, all TLR are expressed in the healthy nasal mucosa as well as in cases of CRS [44]; however, the expression of TLR2, TLR4, and TLR9 receptors and their effector molecule NF-κB in the mucosa of CRS patients is higher compared to healthy people [41], [44], [45], [46]. Disturbed expression of those receptors may contribute to the development of CRS [47].

Intracellular PAMPs, such as for example muramyl dipeptide (MDP) and peptidoglycan are recognized by NOD-like receptors. NOD-like receptors are expressed in the epithelial cells of the nose and the paranasal sinuses [48]. Epithelial cells further express protease-activated receptors (PAR) [49] that are activated by different endogenous and exogenous proteases, including those that are associated with bacteria, fungi, and allergens [50]. The activation of PAR also induces the NFκβ signaling pathway [51]. Proteases of *Staphylococcus aureus* activate PAR-2 and lead to a release of interleukin (IL)-8 [49], [52]. In cases of chronic airway diseases, epithelial cells express more PAR2 than under healthy conditions [49]. 

#### 2.2.4 Indicators of cellular death: damage-associated molecular patterns (DAMPs)

Beside pathogen-associated molecular patterns (PAMPs), cells also recognize cellular damages because of their damage-associated molecular pattern (DAMPs). The combination of foreign material plus cellular damages triggers a reaction of the innate immune system and activated the adaptive immune defense or contributes substantially to its manifestation [53].

In addition to actively secreted inflammatory messengers, recently also those messengers are in the focus of scientific attention that are part of the endogenous intracellular or extracellular matrix of the host and can be released passively into the extracellular space. When released, the original functions of those components change so that they modulate inflammatory reactions under pathologic circumstances in their new environment. In analogy to the PAMPS, those molecules were named DAMPs [54], [55], [56].

Intracellular molecules with extracellular DAMP function are released either passively by damaged cells during an inflammatory or infectious event or actively by certain cell types via so-called alternative secretory pathways because per definition the leader sequence for secretion is missing [56]. DAMPs from the extracellular matrix of the host can be released in cases of damage or by regulated shedding mechanisms [57]. Currently, more than 40 molecules have been classified as DAMP. They exert their effect through activation of at least one toll-like receptor (TLR) or other members of the PRR family, for example via “scavenger receptors” (e.g. CD36) or specialized receptors such as RAGE receptor (receptor for advanced glycation end products) [57]. Several DAMPs also bind to ECM structures like heparan sulphate proteoglycans [56], [57] (Table 2 (Tab. 2)).

Concerning the upper airways, up to now from all known DAMPs and their respective receptors, only the multi-ligand receptor RAGE has been examined intensively [58]. RAGE is a cellular surface protein that is part of the immunoglobulin superfamily and that is made responsible for different inflammatory reactions. RAGE is expressed in full length as membrane-bound receptor (mRAGE), but it also appears as two soluble forms without transmembranous or cytoplasmatic domain; together they are called “sRAGE”. sRage originating from alternative mRNA splicing is classified as “endogenous secretory RAGE” or esRage while RAGE occurring from proteolytic splitting of mRAGE is called cRAGE; this process is initiated by metalloproteinases (MMPs) and ADAMs (A Disintegrin And Metalloproteinases) [59], [60], [61].

mRAGE binds to several ligands, for example to AGE products (advanced glycation end products), HMGB1 (high mobility group box 1), members of the S100/Calgranulin family, the Integrin Mac-1, and to ECM structures such as heaparan sulphate proteoglycans (HSPGs) [62]. sRAGE, on the other hand, seems of act often as kind of a bait receptor with inflammation-inhibiting properties by “intercepting” RAGE ligands of mRAGE surface receptors that transduces inflammatory reactions via the transcription factor NF-κB [63], but it can also promote the inflammation [64]. For mRAGE, too, certain inflammation-inhibiting characteristics have been described [65].

Under normal physiological conditions, RAGE is expressed to a high degree in the lungs – in contrast to many other tissues [65], [66]. In humans, RAGE is also highly expressed in the upper airways under normal physiological conditions and in cases of chronic inflammations it is subject to differential regulation. In CRSsNP, the sRAGE protein quantities are increased whereas the quantity of mRAGE is reduced compared to the control groups. In CRSwNP, the sRAGE and mRAGE protein quantities are reduced in the tissue [58] which is due to an imbalance of metalloproteinases and their natural inhibitors (TIMPs) [67].

Because of the relative lack of collagen in CRSwNP and the excessive collage production with thickening of the collagen fibers in the extracellular matrix (ECM) in CRSsNP [21], [67], the fibrotic tissue has higher quantities of sRAGE than pseudocystic tissue of CRSwNP patients because sRAGE bind to those structures in the long term [66], [68]. In CRSwNP patients, the increased colonization incidence of *Staph. aureus* may contribute to further reduction of sRAGE [69] because *Staph. aureus* induces the release of sRAGE from the ECM [58]. The higher amount of ECM-associated sRAGE in CRSsNP induces an inflammation that is polarized in direction of Th1 [58]. RAGE participates in the differentiation of T cells to a Th1-phenotype; if RAGE is missing, the production of Th1 cytokines is reduced while more Th2 cytokines are produced [70]. Additionally, in CRSwNP a higher amount of protein is found of eosinophil-cationic protein ECP that degrades non-ECM-associated sRAGE so that a reorientation of T helper cells in direction of Th1 is inhibited [58].

Calcium-binding proteins S100A7 (psoriasin), S100A8 (MRP8 or calgranulin A), S100A9 (MRP14 or calgranulin B) and their heterodimer types of S100A8/A9 (calprotectin) have been examined in CRS patients because of their antimicrobial effect [28], [37]. S100 proteins have the typical properties of DAMPs when they are released into the extracellular milieu where they activate TLR4 [71], [72] and possibly also RAGE [73]. In comparison to healthy people, the expression of S100A7, S100A8, and S100A9 mRNA is reduced in patients with CRSsNP and CRSwNP [74]. S100A7 proteins are reduced in the epithelium and in glandular structures in CRSwNP and also the nasal lavage fluid of those patients shows low quantities of S100A7 [37]. In comparison, the amount of heterodimer S100A8/A9 proteins is extremely increased in the polypous tissue because of the higher infiltration of neutrophils, which is a main source of S100A8/A9 proteins [37]. These results indicate that secreted S100A7 and S100A8/A9 proteins from epithelial and/or glandular tissue play a role as antimicrobial substance in the paranasal sinuses and allow the hypothesis that reduced quantities are a possible key to CRS pathogenesis.

Fibronectin is a macromolecular glycoprotein of the extracellular matrix that is very important in the context of wound healing, cell adhesion, matrix formation, protein secretion, cell differentiation, cell cycle progression, and mitogenous signal transduction [75], [76]. It is also associated with immune processes that occur via activation of TLR4; it then has the effect of DAMP [77]. Fibronectin is also considered as target molecule of a high number of bacterial proteins, the bacterial fibronectin binding proteins (FnBPs) [78]; it mediates the adherence of *Staph. aureus* to the extracellular matrix and to the surface of host cells, for example to endothelial cells, epithelial cells, and fibroblasts, through which *Staph. aureus* can enter into the cytoplasm of those cells [78], [79]. Inside the cell, *Staph. aureus* is protected against antibiotics and the immune reactions of the host [79] and thus it can survive which in view of the increased fibronectin expression [39] and the high colonization rate with *Staph. aureus* explains the intramucosal presence of bacteria especially in CRSwNP patients [69].

#### 2.2.5 Lymphoid cells of innate immune defense (ILCs, innate lymphoid cells)

Epithelial cells in the airways produce numerous inflammatory cytokines as normal reaction of the stimulation of PAMP and DAMP receptors [51], among those IL-1, tumor necrosis factor α (TNF-α), type 1 interferons (IFN-α/β), granulocyte macrophage colony stimulating factor (GM-CSF), eotaxins, RANTES (regulated upon activation normal T-cell expressed and secreted), IFN-γ-inducible protein 10 (IP-10), IL-6, IL-8, the growth-regulated oncogene α (GRO-α), monodansylcadaverine (MDC), stem cell factor (SCF), TARC (thymus and activation regulated chemokine), monocyte chemotaxis protein 4 (MCP-4), B cell activating factor (BAFF), osteopontin, IL-32, and Th2 immune reaction associated cytokines such as IL-25, IL-33, and TSLP (thymic stromal lymphopoietin) [26], [49]. Those cytokines do not only cause tissue swelling, vascular dilation, and other characteristics of inflammations, but they often have also chemotactic properties, by which they attract different leukocytes, mast cells, neutrophils, dendritic cells, and lymphocytes to the locus of the inflammation. Also cytokines produced by epithelial cells play a major role in the polarization of dendritic cells and thus influence the type of T cell reaction on antigens [80]. Apparently, there are very special epithelial cell cytokines (IL-25, IL-33, and TSLP) that induce the polarization of dendritic cells and the subsequent T cell differentiation on mucosal antigens [80]. Those cytokines have the particular ability to influence the differentiation of T-cells in direction of Th2-profile as it is known from CRSwNP. Presumably, epithelial cells do not only contribute significantly to the mediation of reactions of the innate immune defense but also influence in this way the subsequent adaptive immune defense. Currently it cannot be excluded that the etiology and pathogenesis of CRS are based on primary variations of the reaction pattern of epithelial cells.

Lymphoid cells of the innate immune defense (innate lymphoid cells, ILCs) have important effector and regulation functions in the innate immune defense and remodeling of tissue, however, they do not perform receptor rearrangement (this property is termed “lineage negative”). ILCs are classified into three categories based on the characteristic patterns of the cytokines they produce and the transcription factors that are necessary for their development and function analogous to the T helper cells: Group 1 ILCs (ILC1) produce interferon γ and depend from the transcription factor Tbet, while group 2 ILCs (ILC2) can produce Th2 cytokines such as IL-5 and IL-13 and therefore need the transcription factor GATA3. Group 3 ILCs (ILC3) also comprise lymphatic tissue inducing cells. ILC3s can produce IL-17 and/or IL-22 and require RORγt as transcription factor. In this regard, ILCs are very similar to T helper cells, however, they are part of the innate immune defense and according to the current knowledge they form the link between epithelium and T cell compartment [81].

CRTH2 and ST2 (IL-33 receptor)-positive ILC2s have been detected in healthy human lungs; however, it is currently unclear which functions ILC2s have in humans in comparison to Th2 effector cells (that both produce IL-5 and IL-13) in the context of pulmonary hemostasis. Accordingly, CRTH2+ILC2s have been identified in CRSwNP tissue [82]. TSLP – an ILC2-activator produced by epithelial cells – is expressed to a high degree in epithelial cells of CRSwNP tissue in patient with asthma [83]. Only little is known about the function of other ILC subgroups in the airways.

In contrast, lipoxin A4, a lipid mediator with inflammation-resolving properties, via the inhibition of IL-13 production of ILC2s [84]. LXA4 also causes the number of eosinophils to decrease by favouring the NK cell mediated apoptosis of those cells. ILC2 localizes in the airways near the mast cells that release prostaglandin D2 (PGD2) which increases the IL-13 production of ILC2s via the PGD2 receptor CRTH2. Thus the activity of ILC2s is regulated by the balance between LXA4 on the one hand and PGD2 on the other hand, whereas both LXA4 and PGD2 are increased in CRSwNP. However, this balance is again disturbed in cases of aspirin sensitivity [85], [86].

#### 2.2.6 Dendritic cells and macrophages

Dendritic cells (DCs) are the link between innate and adaptive immune defense on mucosa. In the airways, several functional subgroups of DCs have been described that differ with regard to their stage of maturity and the expressed combination of PRRs [87]. DCs recognize antigens and process and present them to antigen unversed or “naïve” T cells. Because of these functions, DCs play a key role in the fine adjustment of immune reactions and favor the production of Th1, Th2, or Th17 reactions or regulatory T cells (Tregs) [80]. In the nasal mucosa, 4 different DC subgroups could be identified: 2 myeloid DC populations (mDC) that express either BDCA1 (Blood Dendritic Cell Antigen 1) or BDCA2, a plasmacytoid population of dendritic cells (pDC) that is BDCA1 and CD123 positive (CD = cluster of differentiation), and BDCA1 and CD207 positive Langerhans cells [88]. In CRSwNP tissue where the inflammation pattern is polarized in direction of Th2, an increased number of mDCs and pDCs could be found [89]. This distribution pattern of DCs could contribute to the persisting airway inflammation in cases of CRSwNP.

**Figure 2:**
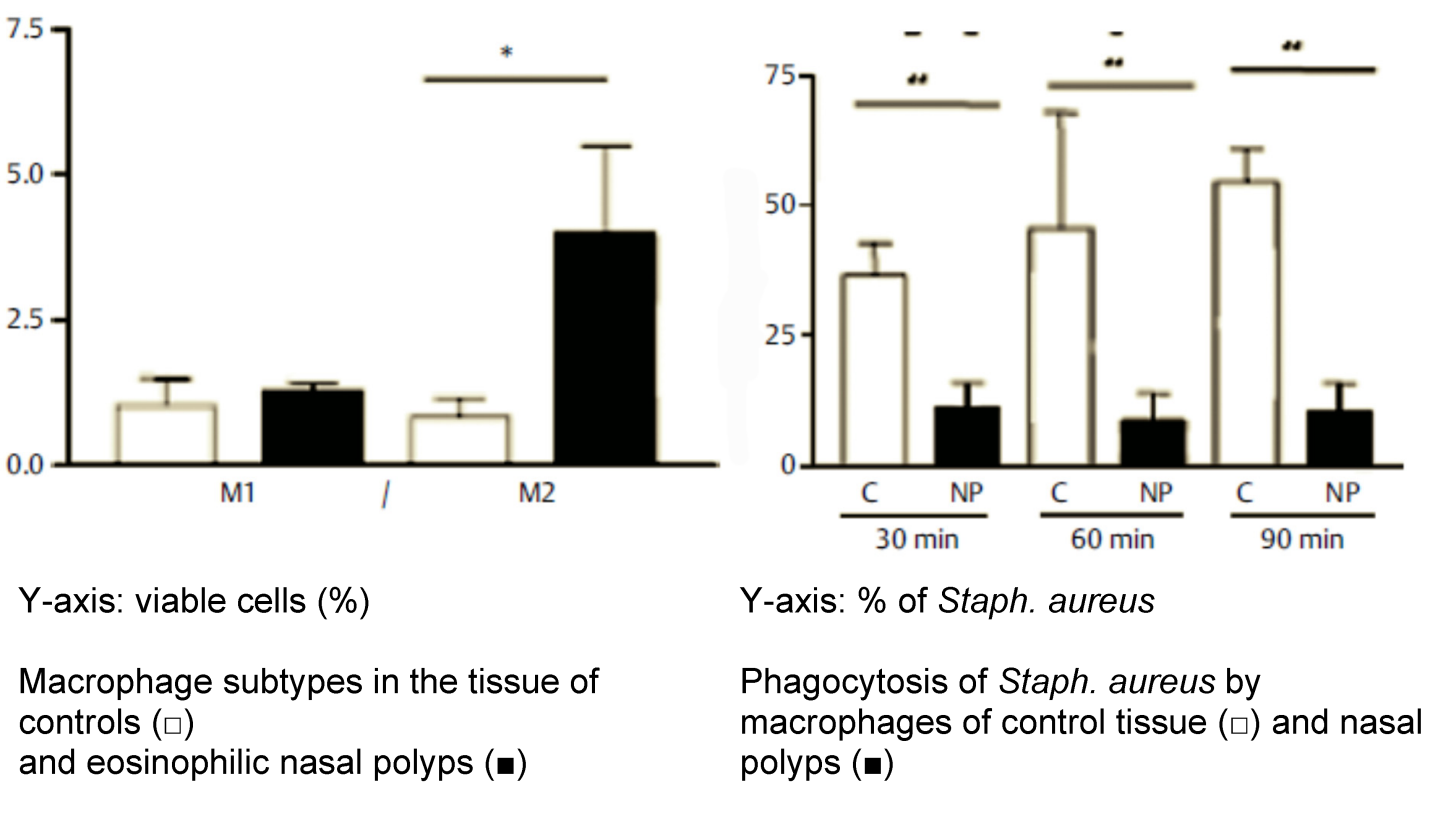
Macrophage subtypes in healthy control mucosa and tissue of eosinophilic nasal polyps: increase of M2 macrophages having significantly limited phagocytosis activity

Macrophages are cells of the innate immune defense that have various functions, as for example to remove particles, to primarily react on pathogens, to maintain tissue hemostasis, to coordinate the adaptive immune defense, to develop inflammation and tissue repair [90]. In accordance to the factor macrophages come in contact with, they polarize to a classically activated inflammatory M1 phenotype or to an alternatively activated M2 phenotype [91]. M1 macrophages express inflammatory cytokines to a high degree such as IL-1β, IL-12, IL-23, and TNF as well as effector molecules as nitric oxide. They contribute to the induction of Th1 reaction and inhibit the survival of pathogens by destroying them effectively inside the cells [92]. However, we could observe that macrophages in IL-5 expressing CRSwNP tissue were mainly M2 polarized and had a high expression of CD206 [93]. Remarkably, M2 macrophages were not able to perform phagocytosis nor to destroy germs like *Staph. aureus*, and this circumstance promotes the intracellular survival of those germs (see later). Furthermore, M2 macrophages from nasal polyps release high quantities of the chemokine ligand 18 (CCL18) which has a chemotactic effect on DCs, naïve T cells, and Th2 cells; all those cells contribute to the pathogenesis of CRSwNP [94] (Figure 2 (Fig. 2)).

### 2.3 Adaptive immune defense: T cells as central components

The adaptive immune defense consists on the one hand of T cells that are especially targeted for this challenge. They may be subdivided into several groups according to the released cytokines and their functions. On the other hand there is the B cell response that is targeted to the production of specific antibodies. The resulting T cell response should be subject to the regulation of T regulatory cells; if not, persisting inflammation occurs. Other bystander cells such as eosinophils and mast cells further modify and amplify the inflammation process, in particular in cases of Th2 reaction. Such cell components sum up the processes that finally lead to most severe diseases of the mucosa, mainly Th2 characterized.

#### 2.3.1 T helper cell patterns

While CRSsNP appears as an only moderate, mainly neutrophil Th1 polarized inflammation, CRSwNP is characterized beside the neutrophil component by a moderate to high eosinopilic Th2 polarized inflammation, at least in Caucasians [95]. Presumably, the reduced TGF-β expression in the CRSwNP tissue contributes to a deficit of Tregs [13], [21], [96] like the strong expression of suppressor of cytokine signaling 3 (SOCS3) which is further enhanced by the reduced migration of regulatory T cells. SOCS3 and Foxp3, which is an important transcription factor of T regulatory cells, is co-expressed in Tregs; SOCS3 regulated Foxp3 expression probably via phosphorylation of STAT3 [96]. The relative deficit of Tregs could be the reason for the inability to suppress the eosinophilic inflammation in CRSwNP patients. In Caucasians, more than 80% of the polyps express a Th2 profile with clear expression of interleukin-5 protein and consecutive tissue eosinophilia whereas this profile is found in less than 20% of all central Chinese CRSwNP patients. Instead, there are Th17 cells that induce a mainly neutrophil inflammation reaction [97].

An extensive flow cytometry of the T cell subtypes was performed in the affected and healthy nasal mucosa in CRS patients [98]. Most of the T cells in the nasal mucosa were CD45RA negative and had an effector phenotype, whereas T cells in the blood mostly have markers of naïve T cell phenotypes. Both in the healthy and in the affected nasal mucosa, a heterogeneous population of T helper cells was present: Th1, Th17, Th22, and Tfh (follicular helper) cells were found. A certain plasticity of the T helper cells could be revealed because for example the Th17 cell population produced not only IL-17, but also IFN and IL-22. The reason for this heterogeneity among the effector T cells might be associated with their protector function, i.e. according to the type of the entering microorganisms, several optimal immune reactions should be possible. The results show that the T cell profile of CRSsNP specimens only differs little from the control specimens which confirms previous findings that CRSsNP is rather a remodeling process than a specific inflammatory reaction [21]. It was remarkable that Th2 cells could only be identified in CRSwNP specimens while asthma patients had the highest number of cells. This pattern correlates with protein data of homogenized tissue specimens after multiplex cytokine analyses [99]. Even if IFN gamma is the most important intracytoplasmatic cytokine in T cells, as protein only low concentrations can be detected in tissue homogenates; apparently IFN is not spontaneously released under basic conditions whereas high concentrations were found after stimulation in the cell free supernatant. IL-5, however, is only expressed by relatively few CD4 positive T cells, but regularly released spontaneously by a CRSwNP endotype; this spontaneous release can be significantly increased by intramucosal *Staph. aureus* germs (see later).

Beside CD4 positive T cells, there is a larger population of T cells in the nasal mucosa that belong to the CD8 cell line and that are the main source of IFN gamma. CD8 positive cytotoxic T cells (Tc cells) express high quantities of Fas ligand that may induce apoptosis in other cells. Another important function of IFN producing CD8 positive T cells (so-called Tc1 cells) is their ability to inhibit IgE response.

**Figure 3:**
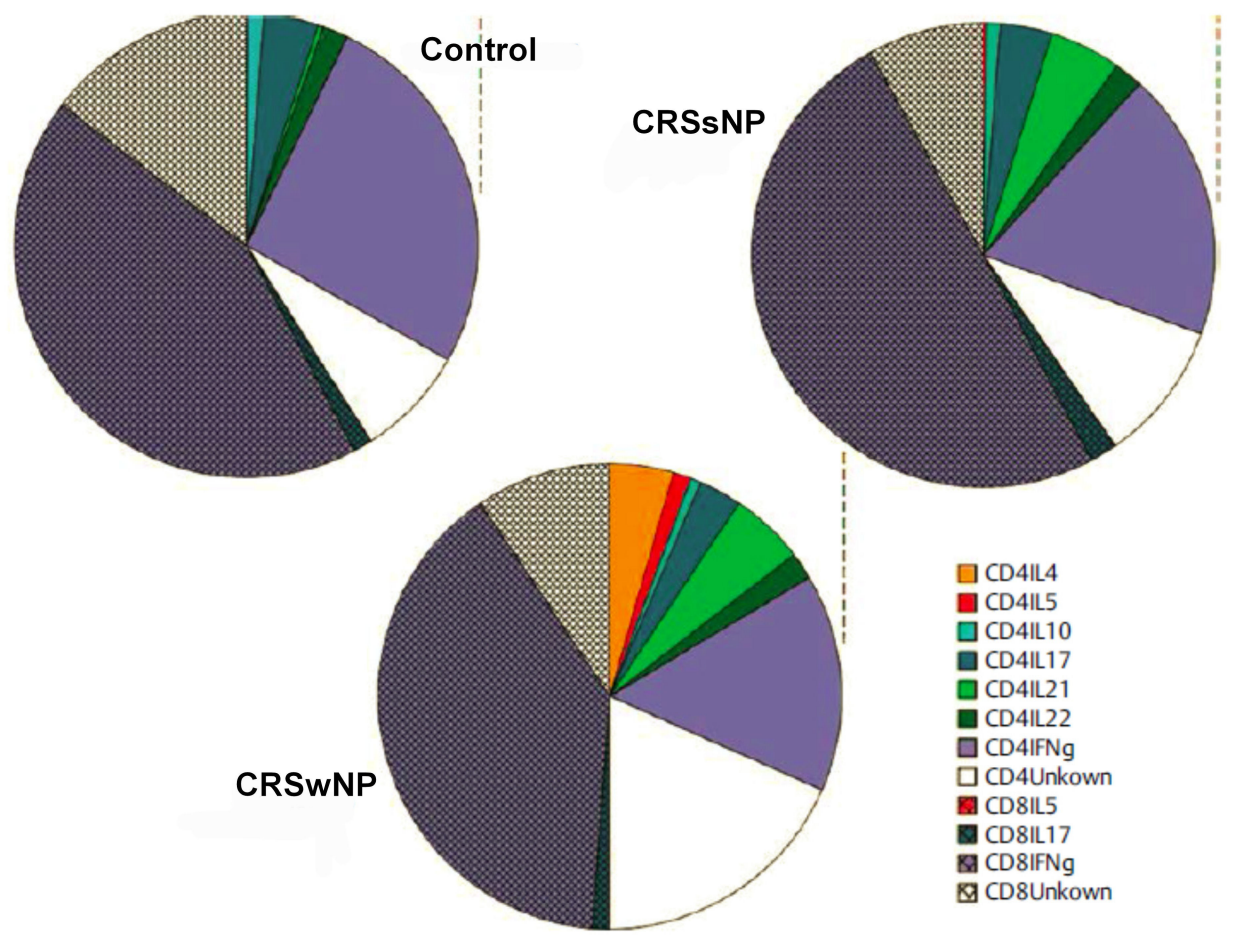
T cell pattern after stimulation and inhibition of the release of cytokines by Brefeldin. Only in cases of nasal polyposis, the T helper cell 2 faction is obvious (IL-4 and IL-5 producing T cells).

The presence of Th2 cells and the expression of IL-5 is associated with eosinophilia, an increased ECP (eosinophil cationic protein) and the total amount of IgE in the mucosa, independent of the atopic status of the patients [97]. The percentage of IL-5 positive nasal polys in Europe amounts to about 85% of all CRSwNP while the percentage in Central Asia, for example in China, is significantly lower with 15% [97]. IL-5 is important for the survival and activation of eosinophils and can be produced by lymphocytes, mast cells, and the eosinophils themselves [100]. The important role of IL-5 for the survival of eosinophils in nasal polyps was revealed in ex vivo trials with human nasal mucosa with neutralizing monoclonal anti-IL-5 antibodies that induced apoptosis in eosinophils and significantly reduced the synthesis of IL-5 and thus also the number of eosinophils. Based on this observation, we performed a pilot projects and studied the possibility to treat severe nasal polyposis with a humanized monoclonal antibody against IL-5 (Mepolizumab) [101]. Two single intravenous injections of 750 mg Mepolizumab led to a significantly improved nasal polyp score in comparison to a placebo group and to a reduction of the polypous mass revealed in computed tomography. This proof-of-concept study confirms the role of IL-5 in CRSwNP and proves the significance of biomarkers for the prognosis and thus the optimized patient selection (Figure 3 (Fig. 3)).

#### 2.3.2 B cells and local immunoglobulin production

In CRSwNP, the total number of B and plasma cells such as the relation of plasma cells and B cells is partly much increased in comparison to control groups which might be a hint to an active B cell compartment in this disease, but not in CRSsNP [102]. In cases of eosinophilic CRSwNP, the local IgG and IgE concentrations, and important markers of a local immunoglobulin class switch are as well increased in comparison to allergic rhinitis and healthy people [103]; also this phenomenon is not observed in CRSsNP. During the immunoglobulin class switch, recombination events occur (class switch recombination, CSR) typically via IgG4 to IgE. In up to 30% of the B cells, plasma cells, and T cells in nasal polyps a repeated expression of RAG1 and RAG2 (recombination activation genes) can be revealed that are necessary for receptor revision [103]. Furthermore we were able to identify the up-regulation of activation-induced cytidine deaminase (AID), which is a factor indicating as well an active CSR and somatic hypermutation of immunoglobulins [104]. In CRSwNP, the concentrations of RAG1 and RAG2 mRNA is increased and correlates with the severity of the inflammation and the presence of *Staph. aureus* enterotoxin (superantigen)-specific IgE in the mucosa of nasal polyps. Those results prove a local receptor revision and the class switch to IgE and the differentiation of B cells to IgE secreting plasma cells in polypous tissue of CRSwNP without obligatory involvement of other lymphoid structures as for example lymph nodes. In tissue homogenates of CRSwNP the concentrations of important markers of Th2 induced inflammatory processes including IL-4 are significantly increased; IL-4 stimulated the synthesis of the ε-germ line transcript (GLT) that is used during CSR to IgE [103]. In CRSwNP, the concentrations of ε-GLT are significantly increased. The same is true for the B cell activating factor of the TNF family (BAFF) [105], which stimulates the development of follicles, and the B lymphocyte-induced maturation protein (BLIMP). Both BAFF and BLIMP are involved in the differentiation of B cells to plasma cells [106], [107]. Finally, we could reveal the presence of Iε-Cc class switch transcripts by means of gel electrophoresis and southern blotting and confirm it by sequencing of 339 bp long cDNA sequence, which unequivocally proves the local class switch in CRSwNP tissue [103]; a local IgE synthesis was previously shown in allergic rhinitis [108].

The proof for the local receptor revision was given by the up-regulation of RAG1 and RAG2 in B cells, plasma cells, and T cells. Indeed, the percentage of those cells that express RAG proteins in CRSwNP is similar to tonsillar B cells (15–26%) and significantly higher than in circulating B cells (<5%). Relevantly high percentages of B cells (20–30%) can also be found in target organs of autoimmune diseases. Recently it could be shown that both BAFF and the autoantibody are increased in nasal polyps and possibly play a role in the pathogenesis of CRSwNP [109].

**Figure 4:**
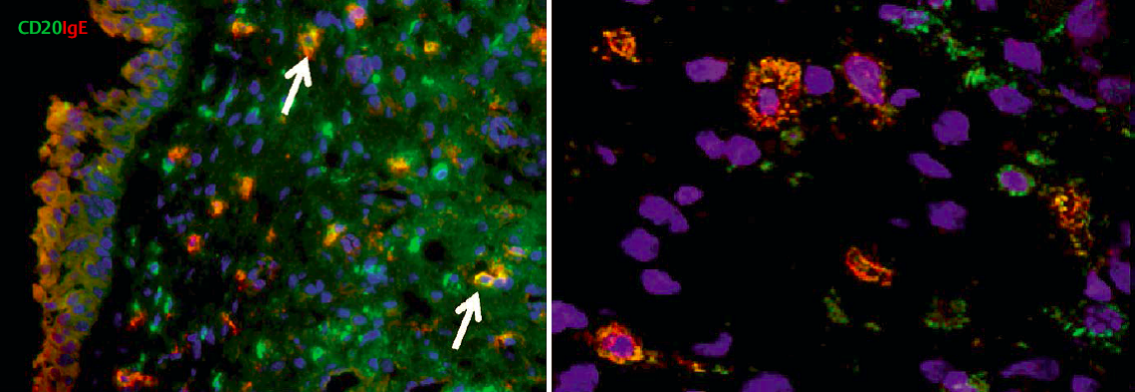
IgE positive B cells and IgE producing plasma cells in nasal polyposis

*Staph. aureus* enterotoxin IgE was found in about 30% of CRSwNP patients, but not in the nasal mucosa of control groups. In the SE IgE positive polyps, the quantities of IgE, ECP, but also RAG1 and RAG2 were significantly increased than in SE IgE negative polyps which may be a hint to a direct stimulation of the RAGs by *Staph. aureus*. The correlation between RAG expression and a confirmed contact with *Staph. aureus* enterotoxins proves convincingly that reactions have been stimulated in the local germinal center and the polyclonal IgE expression by superallergenes [103]. We have already shown that local SE specific IgE directed against enterotoxins and polyclonal IgE resulting from the activation of plasma cells may contribute to the form and persistence of inflammation in CRSwNP; such IgE antibodies are active against several 100–1,000 different inhalative allergens and against allergens of *Staph. aureus* itself and cause a degranulation of mast cells after exposition [110]. Those results correspond to the fact that the treatment of CRSwNP with anti-IgE antibodies (Omalizumab) clearly reduces the size of the polyps and the symptoms of the disease (Figure 4 (Fig. 4), Table 3 (Tab. 3)).

#### 2.3.3 Mast cells and eosinophilic granulocytes

Mast cells often occur in the nasal mucosa and have a physiologic function in the context of innate immune defense and wound healing. The activation of mast cells leads to the release of already present granules containing histamine, serotonin, proteoglycans, and serine proteases and to the de novo synthesis and release of different eicosanoids, chemokines, and cytokines. Prostaglandin D2 from mast cells was correlated with the recruiting and activation of Th2 lymphocytes in nasal polyps [86]. The stem cell factor (SCF) released from epithelial cells might substantially contribute to the recruiting of mast cells in nasal polyps [111]. In cases of allergies, the mast cell degranulation is initiated via an antigen-induced linkage of IgE molecules that are bound to highly affine receptors on the mast cell surface. However, there are other IgE-dependent and IgE-independent processes inducing, enhancing, and maintaining degranulation [112]. In particular, studies with explanted polyps have shown that a mast cell degranulation can also be triggered by other mechanisms including protein A (SpA), which is a surface protein of *Staphylococcus* [113]. Interestingly, it could not be definitely clarified if the number of mast cells in CRSwNP was increased in comparison to CRSsNP or control tissue [114]. Functional studies, however, allow the hypothesis that mast cells are much more active in nasal polyps and in vivo react more sensitively on external triggers [113]. With regard to the massive polyclonal IgE production, which is typical for CRSwNP, and to the possibility of direct activation of mast cells by means of special proteins of *Staph. aureus*, it is highly probable that in cases of CRSwNP a persisting activation of mast cells occurs. The inhibition of the continuous mast cell activation by innovative approaches might contribute to clarifying the significance of those cells in persisting inflammatory processes.

Eosinophils are circulating granulocytes that are responsible for immune defense at mucosal surfaces, primarily against multicellular parasites, but also for the remodeling and the repair of the mucosa. Their high amount in the mucosa of the airways in CRSwNP is associated by some authors with a certain origin of a disease, especially with the infection with fungal spores, however, there is still no final proof. The degree of eosinophilia in CRS is independent from the simultaneous presence of allergic rhinitis or fungal sinusitis which possibly allows concluding overlapping pathophysiological processes. In addition, the degree of tissue eosinophilia in CRSwNP correlates with the severity of the disease, the diagnosis of aspirin sensitivity, the occurrence of comorbid asthma [115], and the necessity of surgical revision [116]. At the same time, eosinophilia is not imperatively associated with CRSwNP; in 85% of the polyps of Asian patients as well as in about 15% of the polyps of Caucasian patients, the cellular pattern is not eosinophilic, but neutrophil. Instead of the expression of IL-5, IL-17 and IFN-γ are found as well as corresponding transcription factors [97].

For eosinophils, the chemokines RANTES, eotaxin 1-3, MCP 1-4 are relevant that are all mainly secreted by nasal epithelial cells and exercise their functions via the receptor CCr3 [117], [118], [119]. Also dendritic cells and macrophages may contribute to the production of eotaxin and other CCR3 ligands. The Th2 cytokines IL-4 and IL-13 play an important role in the induction of chemokines via STAT6 and NF-κβ. GM-CSF and IL-5 induce an enhanced migration and adhesion of eosinophils in the tissue of nasal polyps and favor the survival of those cells; IL-5 is an important priming and survival factor for eosinophils and antibodies against IL-5 induce the apoptosis of eosinophils in CRSwNP tissue [100], [120]. In summary, the process of recruiting, activation, and differentiation of eosinophils in CRSwNP is mainly promoted by Th2 cells and the cytokines released by them. There are sound hints that *Staphylococcus* superantigens favor eosinophilia in polypous tissue by enhancing the local release of Th2 cytokines by acting on the Th2 cells [99], [113], [115]. Recently, there were even hints that also *Staphylococcus* biofilms might favor eosinophilia in CRS independent from the polypous status or from super antigens [121]. The mechanism of this possible effect is unclear. Factors as for example IL-33, TSLP, and IL-25, which activate ILC2, might induce the production of IL-5 and thus influence the immune reaction in direction of a Th2 profile (Len Feng, unpublished data).

In the literature it is abundantly described that glucocorticosteroids can inhibit the recruiting, survival, and activation of eosinophils in CRS [122]. A recently performed double-blind trial with oral corticosteroids confirmed the clinical, however short-term, effectiveness on the nasal polyp score and the reduction of IL-5 and ECP in nasal secretion [123]. Both IL-5 and its soluble receptor are increased in eosinophilic nasal polyps [124]; this constellation locates the cells in the tissue. Studies with monoclonal antibodies against IL-5 showed a clear clinical effect while the amount of eosinophils in the blood and the mucosa significantly decreased after this kind of therapy [101].

Also neutrophils are circulating immune effector cells with well described functions in the early phase of defense, phagocytosis, and killing of extracellular microorganisms. In CRS, especially the chemokine IL-8 that is partly released from nasal epithelial cells as reaction on PAR-2 stimulation seems to trigger the recruiting of neutrophils [49]. The role of neutrophils in CRS is still unclear; in the mucosa of mucoviscidose patients, their amount is extremely increased together with the concentration of IL-17 [125].

In CRSsNP and CRSwNP, the degree of infiltration with neutrophils can be compared, in cases of cystic fibrosis it is much higher; in contrast, the infiltration with eosinophils is significantly higher in CRSwNP than in CRSsNP; each eosinophilic polyp also has a relevant number of neutrophils. Polyps of Chinese patients were non-eosinophilic or neutrophil in 85% of the cases [97]. However, this pattern may change so that the inflammation achieves a rather eosinophilic character in the course of time, accompanied by the presence of eosinophilic mediators, IgE and *Staph. aureus* in the mucosa [126].

#### 2.3.4 Factors contributing to the resolution of inflammations (resolvins)

Not only inflammatory processes, but also their resolution are active processes where numerous lipid mediators are involved. The recently discovered inflammation resolving resolvines of the E and D series, protectins, and maresins have a strongly inhibiting effect on human cells. This is why those three mediator families and their acetylsalicylic acid triggered forms were called specialized pro-resolving factors (SPMs) [127]. Arachidonic acid (AA) and omega-3 fatty acid – the essential eicosapentaenoic acid (EPA) and docosahexaenoic acid (DHA) – are substrates for the biosynthesis of highly inflammation inhibiting or inflammation resolving mediator substances. As it is a relatively young field of research requiring highly sophisticated measuring techniques, there is only little known about the presence and role of those SPMs in the upper airways. However, the pro-resolving mediator substance of lipoxin A4 originating from arachidonic acid has already been measured in patients with CRs [85]. Tissue of nasal polyps from the upper airways of CRSsNP and CRSwNP patients had higher concentrations of LXA4 than healthy nasal mucosa; in contrast, the LXA4 concentration in patients with aspirin sensitivity was relatively reduced in comparison to patients with nasal polyposis without such a sensitivity. Probably, the same pattern could be found in other SPMs: in order to reduce inflammation, the up-regulation of pro-resolving factors in the affected mucosa is induced; this process, however, seems to be disturbed in particularly severe types of inflammation such as NERD so that the inflammation may become persistent. The external substitution of pro-resolvins might be a possible therapeutic approach. 

### 2.4 Remodeling of the mucosa of the paranasal sinuses

Cellular remodeling processes are dynamic processes where the production and degradation of the extracellular matrix (ECM) are balanced and regulated by different mediators among them TGF-β. TGF-β is considered as central factor in the remodeling process in the airways because it attracts fibroblasts, induces their proliferation and up-regulates ECM synthesis (e.g. of collagen). As already described, the protein concentrations of TGF-β1 and 2, the expression of mRNA of the TGF-β receptor (R) I and RIII and the number of activated cells with positivity for pSmad 2 (phosphorylated protein “mothers against decapentaplegic homolog”) (which is a sign for the activation of the cell by TGF-β) is significantly higher in patients with CRSsNP compared to control groups [21]. In CRSwNP patients the TGF-β1 protein concentration, the expression of TGF-β-RII and -RIII mRNA as well as the number of activated pSmad 2 positive cells were significantly lower than in control groups. Those data confirm a relevant difference between these two CRS subgroups with regard to the regulation of TGF-β and its signaling which manifests as a deficient collagen production in CRSwNP and as exceeding collage deposition in the ECM in CRSsNP. The TGF-β signaling also contributes to the regulation of metalloproteinases (MMP) and their natural tissue inhibitors (TIMP; tissue inhibitors of metalloproteinases). The overexpression of MMP7 and MMP9 without compensation by TIMP1 and TIMP4 is associated with a degradation of ECM, the development of vacuoles, and the deposit of albumin in nasal polyps, whereas the up-regulation of TIMP in CRSsNP inhibits edema [67]. Thus, CRSsNP may be characterized as fibrosis while CRSwNP is rather described as edema.

**Figure 5:**
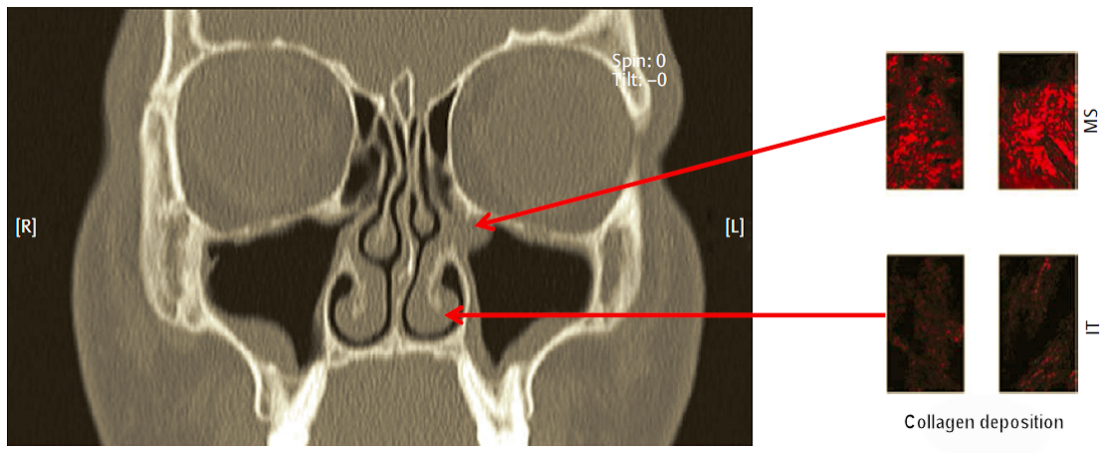
Collagen deposition in the area of the osteomeatal complex in comparison to the inferior turbinate in beginning CRS

**Figure 6:**
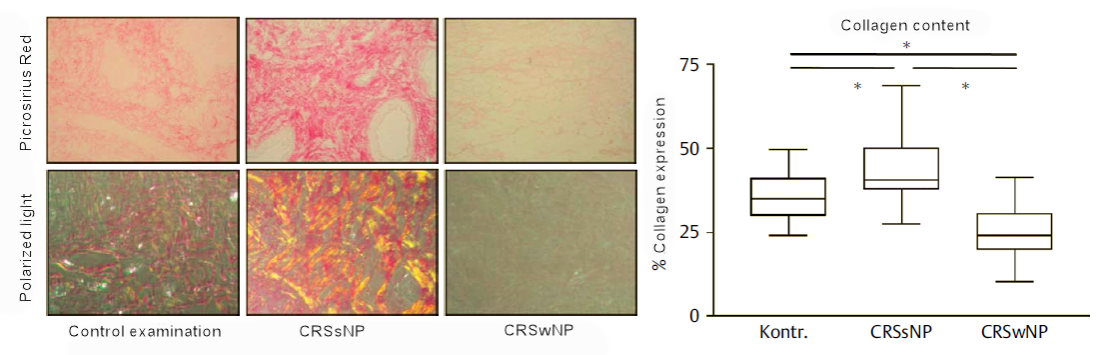
Collagen content and collagen bundles in polarized light in control groups, CRSsNP, and CRSwNP. In contrast to the overproduction of collagen and fibrosis in CRSsNP, there is a deficit of collagen in CRSwNP.

It might be presumed that the tissue remodeling is a consequence of the inflammation; this sequence could be brought in line with the high activation of TGF in the mucosa adjacent to the polyps and with the deposition of albumin in nasal polyps correlating with IL-5 [39]. More recent data, however, allow drawing the conclusion that remodeling precedes the inflammation at least in CRSsNP [128]. In a study where TGF-β and inflammatory cytokines were measured in the different paranasal sinuses of patients in an early stage of CRSsNP the protein concentrations of TGF-β1 were significantly up-regulated in the area of the osteomeatal complex, which is the central access to the paranasal sinuses, and were accompanied by an increased production and deposition of collagen in this area. Th1-regulated and inflammatory cytokines or myeloperoxidase protein as markers of activated neutrophils, however, were not up-regulated in these patients at that time. Those observations emphasize the central role of TGF-β and suggest that the remodeling in CRSsNP occurs independently from the inflammatory processes and precedes it (Figure 5 (Fig. 5) and Figure 6 (Fig. 6)).

### 2.5 Interaction between bacteria (microbiome) and immune defense

According to the current opinion, the detection of pathogenic germs on the nasal mucosa by swab means automatically disease of at least colonization that may lead to disease at any time. Frequently occurring germs of bacterial infections of the upper airways are germs like *Staph. aureus, Hemophilus influenza, Pseudomonas aeruginosa, *and* Moraxella catharralis*. Today it is well-known that the nasal mucosa is always, at any time in life, colonized with hundreds of different germs that – as far as their presence is balanced – guarantee a healthy condition. With the advent of the field of molecular biology, culture independent methods have been developed in order to examine microorganisms based on their genetic patterns. As a consequence, a clearly more complex flora was discovered in the upper airways than it had been suspected until that time. Due to the development of culture independent means it is meanwhile possible to identify species of microorganisms that could not be detected with previous growth methods. Today it is known that the human body is the home of 10–100 billions of microorganisms, an amount that is far higher than the number of body cells. The bacterial flora that has been known only for a short time is called human microbiome. Different regions of the body such as the nasal mucosa, skin, urogenital tract etc. have their own microbiomes encompassing ideally many microorganisms in relatively similar low frequencies (“richness, evenness”). If single germs disturb this balance by proliferation and by suppression of others, a pathologic situation occurs.

Based on the creation of comparing micriobiome profiles in a small cohort of CRS patients, on whom no further details were defined, and healthy test persons, it was assumed that the flora of the paranasal sinuses of CRS patients has a significantly lower multitude of bacteria compared to healthy controls. While on the one hand numerous phylogenetic distinct lactic acid bacteria were missing, on the other hand an increase of the relative incidence of a certain type of *Corynebacterium tuberculostearicum* was observed [129]. The authors drew the conclusion that this germ could be made responsible for the pathogenesis of CRS; for confirmation of its pathogenic role of a certain microbiome constellation in a specific inflammation status, however, multicentric studies in large, well defined CRS populations are required [130]. Additionally, the relationship between microbiome and immune defense in the mucosa could be bidirectional, i.e. the bacteria exercise a certain pressure while on the side of the host an insufficient defense is present, for example because of alternatively activated macrophages, that show a reduced phagocytosis and an insufficient killing of intracellular germs [93]. Scientific examinations on how certain germs influence the immune reaction of the mucosa of the nose and paranasal sinuses, could shed new light on the pathophysiology of CRS and lead to new treatment approaches. The manipulation of the flora or the introduction of specific healthy germs (e.g. lactic acid bacteria) might turn out to be helpful for the treatment of inflammatory diseases and break the dominance of certain germs such as for example *Staph. aureus* or* Pseudomonas aeruginosa.*

#### 2.5.1 Interaction between viruses and bacteria

Common colds rank among the most frequently occurring infectious diseases of humans and are mainly caused by human rhinoviruses, the respiratory syncytial virus (RSV) as well as corona, adeno, and parainfluenza viruses. An adult suffers about 2–5 times per year under colds while the rates of infections in females aged between 20 and 34 years are higher, probably because of infection by their children. Children have 6–8 common colds per year, day care institutions for children represent a relevant risk factor for diseases of the airways in children and their mothers. Because of the fact that certain viruses appear seasonally, a clear dependence from the seasons is observed with higher incidences in fall and winter [131].

Typical initial symptom of a common cold is a sore throat because the virus preferably binds via the intercellular adhesion molecule 1 (ICAM-1) to the tissue of the nasopharynx/lymph, subsequent nasal symptoms are sneezing, nasal secretion of fluid or mucus (rhinorrhea), and nasal obstruction. The infection can extend via the Eustachian tube to the middle ear or via the trachea into the lower airways so that coughing and bronchitis occur. After 10–14 days the symptoms regress spontaneously if no acute postviral rhinosinusitis occurs [1].

Viral infections of the nasal cavity are the most common reason of acute asthma exacerbation in children (80–85% of asthma exacerbations) [132] and in adults (about 80% of asthma exacerbations) [133]. In two third of the cases with proven virus, rhinoviruses are detected. In asthmatics, the up-regulation of ICAM-1 on epithelial cells, a receptor that is used by 90% of the rhinoviruses, might be responsible for the higher sensitivity especially of atopic patients with more frequent infections and more severe and longer lasting symptoms [134]. In vitro studies reveal that rhinoviruses replicate more efficiently in primary bronchial epithelial cells of asthmatics than healthy people. There is a strong correlation between the virus charge and the severity of the symptoms in the lower airways and an increase of the bronchial hyperactivity. In association to the pathogenesis or rhinovirus induced asthma exacerbations, impaired Th1 reactions (IFN-γ and IL-12), that seem to have mainly a protective effect against the replication of viruses, and enhanced Th2 reactions (IL-4, IL-5, and IL-13) are described as origin of more relevant virus-induced asthma symptoms [135].

In adults suffering from mild/moderately severe atopic asthma and in bronchial epithelial cells of children with severe therapy-refractory atopic asthma, a defective induction of type-1 interferon (IFN-β) and type-III interferon (IFN-λ) was found [136]. Actually, patients with severe therapy-refractory asthma had an increased virus replication that correlated negatively with the amount of interferon mRNA. Also a study performed with asthmatic children living in cities revealed that the treatment with antibodies against immunoglobulin E (IgE) could improve the control of asthma, reduce the need of asthma medications, and nearly eliminate the seasonal peaks of exacerbations induced by virus infections [137]. This would mean that the reduced Th1 immune reactions might be based at least partly on an enhanced Th2 immune response.

**Figure 7:**
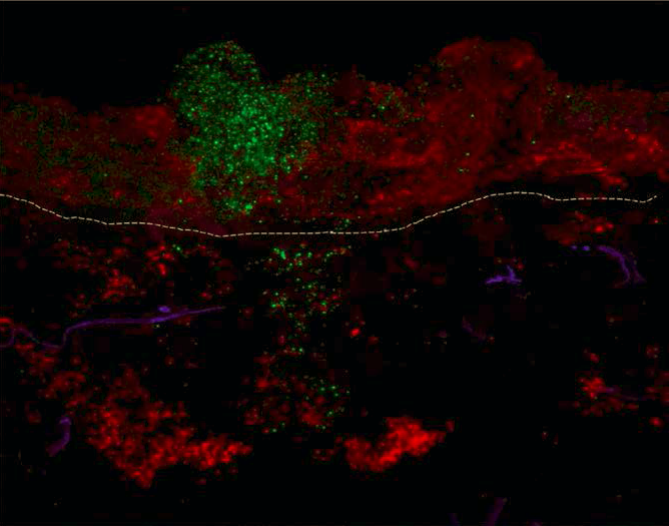
Inferior turbinate, 24 hours after infection with rhinovirus (HRV 16, red) and *Staph. aureus* (green). The basal membrane is marked by the dotted line. *Staph. aureus* uses the epithelial damage caused by the infection with HRV 16 and infiltrated the mucosa.

Actually, unpublished results show that also tissue of nasal polyps in the ex vivo mucosal model respond insufficiently to herpes simplex virus (HSV1) and rhino virus infections; a deficient IFN-γ production and IL-17 release as well as an increased release of TNF-α were the main characteristics of mucosal reaction on a virus infection in CRSwNP with Th2 polarization in comparison to healthy inferior turbinates (Lan Feng, in press). This deficient virus defense could lead to another phenomenon: as virus infections open the doors to bacteria and allow them entering into the mucosa despite an otherwise intact epithelial barrier [138], the probability of bacterial ingress, such as for example *Staph. aureus*, could be increased in cases of frequent virus infections. Because of the fact that an alternative activation of macrophages occurs in a Th2 polarized environment (IL-5 plus CRSwNP), the intruders could avoid the defense mechanisms of the mucosa in such a situation and colonize the mucosa without being phagocytized or killed by macrophages [93] (Figure 7 (Fig. 7)).

#### 2.5.2 The role of Staphylococcus aureus as amplificator of the inflammation

*Staph. aureus* colonizes the nose very frequently; about one third of all Europeans are life-long carriers of coagulase positive *Staph. aureus*. Especially in CRSwNP [69] *Staph. aureus* colonizes the middle nasal meatus and the surface of the nasal polyps. The colonization rates are very high with 67 and 87% of the patients with CRSwNP and asthma or NERD, respectively. *Staph. aureus* does not only colonize the mucosa but produces biofilms adhering at the inflamed and damaged mucosa in order to serve as reservoir for planktonic germs. Every now and again single germs are released from the biofilms that protect, inform, and nourish germs as three-dimensional structures. Those germs re-infect the mucosa [121]. By means of PNA-FISH (a peptide nucleic acid fluorescence in situ hybridization technique) intramucosal *Staph. aureus* can be identified very specifically that is found intracellularly only the tissue of CRSwNP patients [139]. The germ can also be present as “small colony variant”, a sleeping variant with low metabolism, or replicate in epithelial cells of nasal polyps [140]. Those findings confirm the persisting character of *Staph. aureus* infection and indicate its possible role as amplificator of inflammations by means of its immune proteome; beside the superantigens, *Staph. aureus* produces a multitude of proteins that may have an impact on the human immune system.

Manipulation of the immune system means advantages for the germs. We could show that the Th2 inflammation of the nasal polyps favors the alternative activation of macrophages to so-called M2 macrophages while those immune cells are characterized by a reduced phagocytosis and intracellular killing of germs including *Staph. aureus* compared to the usually favored M1 macrophages [93]. This deficiency of the mucosal defense allow *Staph. aureus* to colonize the mucosa and to enhance the Th2 immune reaction via different mechanisms.

*Staph. aureus* synthesizes and secretes numerous immune proteins and enterotoxins, among those the classical enterotoxins (SEs) SEA, SEB, SEC, SED, SEE, and toxic shock syndrome toxin-1 (TSST-1) [141]. SEs, also known as superantigens, are able to activate T cells via an antigen-unspecific binding to the variable β chain of the T cell receptor which again allows a polyclonal activation of a high number of T cells with different characterization; up to 20% of the local T cells can be activated at the same time instead of the usual rate of less than 0.1% which may cause a “cytokine storm” [142]. This cytokine storm can lead to the sudden death of the patient, and in cases of chronic triggering it maintains a severe persisting inflammatory reaction within the tissue. Furthermore, SEs can activate B cells, eosinophils, epithelial cells and other cell populations which enhances the immune globulin synthesis including IgE and the migration of eosinophilic granulocytes in the tissue. The result is a further Th2 polarization; SE-IgE positive nasal polyps have a manifold increased IgE, ECP, and IL-5 compared to SE-IgE negative polyps. Recently, we were able to provide the proof of the local release of SEs and other immune proteins from *Staph. aureus in* the tissue of nasal polyps by means of proteomics (unpublished data). The changes of the innate and adaptive immune response induced by SEs [143] promote the survival of *Staph. aureus*. In an animal model, SEs cannot only enhance Th2 mediated inflammations of the airways but also induce them [144], [145]. The intranasal application of allergens in mice normally leads to development of tolerance; the addition of SEB, however, leads to sensitization and formation of an eosinophilic inflammation reaction of the upper and lower airways [146]. We could show in human mucosa that protein A and SEB from *Staph. aureus* lead to a significant degranulation of mast cells and to the activation of Th2 cells with consecutive release of the Th2 cytokines IL-4, IL-5, and IL-13 [113]. These effects further influence the T cell response in direction to Th2 and thus contribute to a persisting, in particular eosinophilic inflammation of the airways. Furthermore, SEB induces the release of chemokines from epithelial cells which leads to the migration of granulocytes and their activation [147].

Recently, we could also prove that actually intramucosal *Staphylococcus* germs amplify the production of IL-5 in nasal polyps; after killing by antibiotics or *Staphylococcus*-specific bacteriophages, the spontaneous IL-5 production significantly reduced to the level of the polyps that were not colonized by *Staph. aureus* (Bachert, in press).

However, most important is the ability of staphylococcal products like SEA and protein A to stimulate the production of IgE antibodies [148], [149]. This B-cell stimulation and transformation to plasma cells can also be observed in tissue of nasal polyps [150]; in this context accumulation of very high concentrations of IgE in the tissue results while more than 5,000 kU/l IgE were measured in the tissue of nasal polyps [102]. A real local IgE synthesis in polyps may be organized via the formation of follicle-like structure in the nasal polyp and the expression of a series of mediators, that are important for immunoglobulin synthesis, such as AID (activation-induced cytidine deaminase) and BAFF, while the class switch of IgG to IgE and the synthesis of specific IgE antibodies do not require further lymphatic structures like lymph nodes [104], [115], [151]. Recent examinations confirm this hypothesis of a local immunoglobulin synthesis triggered by SEs based on the proof of SE-IgE associated expression of RAG1 and RAG2 [103].

**Figure 8:**
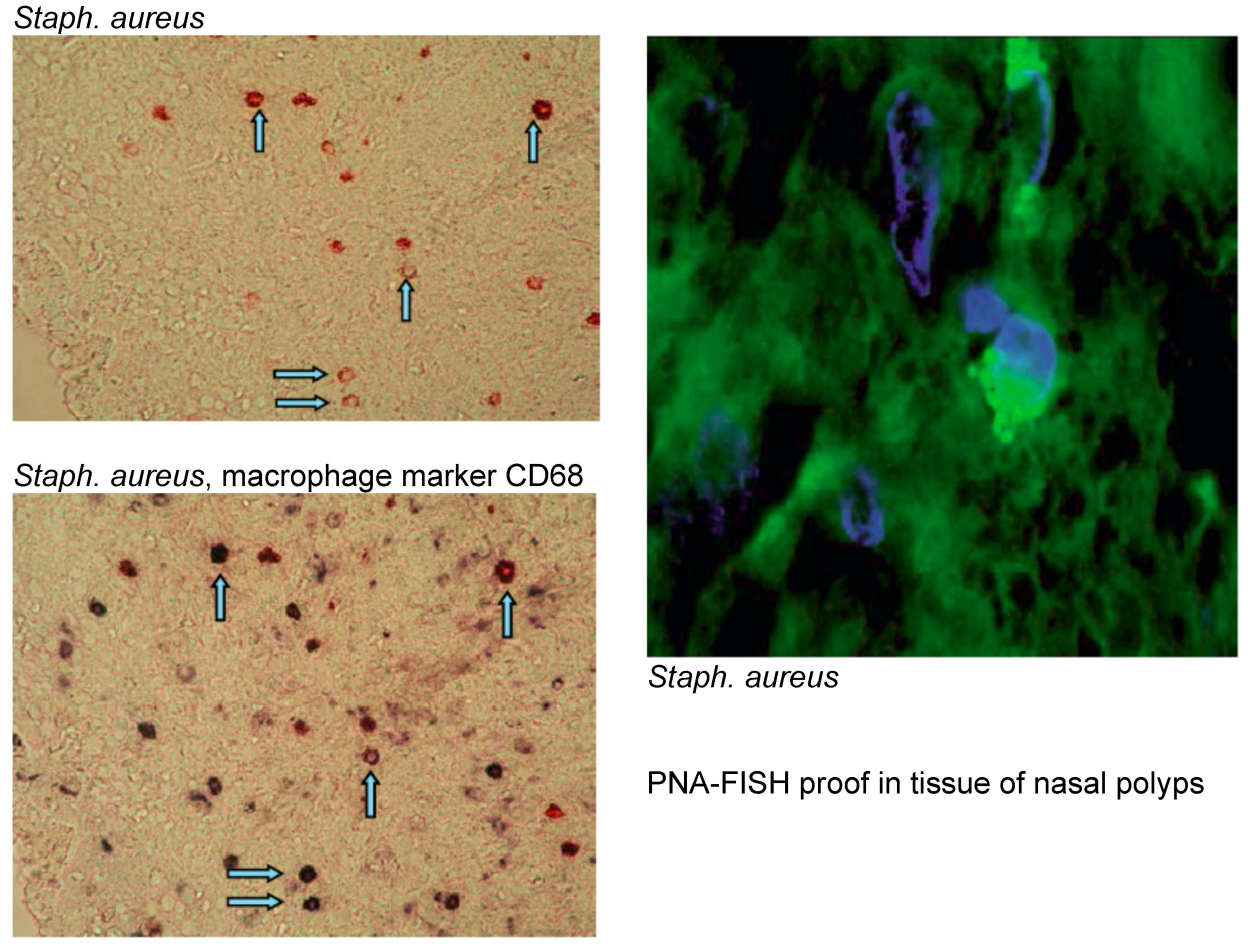
*Staph. aureus* can be localized in macrophages (M2) and identified as single cell in the tissue by in situ hybridization (PNA-FISH).

Specific antibodies in polypous tissue are only a small part (mostly less than 2%) of the total IgE which can only hardly be identified or not identified at all. Nonetheless, this polyclonal IgE is functional. According to the presence of specific IgE antibodies in the tissue, as mast cell degranulation can be triggered by exposure to the corresponding allergen, independent from the presence of the IgE antibody in the serum [110]. This means that skin tests or serum IgE measurements may be negative and nonetheless a local degranulation of mast cells is triggered by allergen contact; the IgE of nasal polyps can be transferred to basophiles and also there it triggers a specific activation of the basophiles (RBL SX38 cells) in tests. This means the proof of the functionality of local polyclonal IgE [110] and these observations can also be transferred to the lower airways (Figure 8 (Fig. 8)).

### 2.6 From sinusitis to asthma

Around 40% of our patients with nasal polyposis also suffer from asthma. The relation between CRS and asthma has recently been proven for Europe in a multi-centric study of the GALEN group [6]. This association applies to the so-called late-onset asthma, however, the patients are mostly not allergic. In a study on the definition of biochemical factors in the tissue of nasal polyps associated with an increased risk for comorbid asthma within the group of CRSwNP patients, it could be observed that IL-5, IgE, and SE-IgE predict asthma. If SE-IgE is present together with a high total IgE, the risk of comorbid asthma is multiplied compared to SE-IgE negative CRSwNP patients [99]. This observation could recently be confirmed in an independent cluster analysis. Two of 10 CRS clusters were SE-IgE positive and had a rate of comorbid asthma of 65–75% in contrast to CRs patients whose comorbidity was less than 10% (Tomassen, in press). Eosinophil but SE-IgE negative CRSwNP patients suffer from asthma in 20–40% of the cases.

Also in patients with severe asthma, independent from the diagnosis of polyposis, SE-IgE positivity is found significantly more often (60%) than in control groups (13.0%, P<0.001) [152]. A logistic regression analysis showed a significantly higher risk of each type of asthma in SE-IgE positive patients (OR 7.2 [95% confidence interval (CI) 2.7–19.1] or of severe asthma (OR 11.1 (95% CI 4.1–29.6)) in comparison to serum SE-IgE negative patients. According to skin test and serum IgE, 21% of the patients with severe asthma were non-atopic on inhalative allergens; thus SE- IgE is a factor of “intrinsic” asthma independent from an atopia. The application of oral corticosteroids, the number of hospitalizations because of acute deterioration of the asthma, and the lung function parameter FEV in 1% were all associated with SE-IgE in the serum [152]. So we assume that *Staph. aureus* plays a major role in the pathophysiology of severe asthma and SE-IgE in the serum marks the involvement of the germ in the disease.

The sensitization against *Staph. aureus* enterotoxins is rather frequent in Europe with a prevalence of nearly 30%. For the first time, this evaluation proved in a large epidemiologic study performed in the population of the EU that SE-IgE is clearly associated with asthma [153]. The results have recently been confirmed by a Korean study group [154]. Another study performed in 240 older patients with asthma (onset of the disease ≥40 years), however, showed that the SE-IgE concentration in the serum of the patients was significantly higher than in control groups. Furthermore, SE-IgE correlated with the total IgE, the eosinophilia in the sputum, with the severity of the asthma, and the diagnosis of CRS (Song, in press). The observations emphasize the role of SE-IgE in non-atopic asthmatics with late onset and indicate the significance of CRSwNP in this patient population.

Even in children and adolescents, associations between SE-IgE and asthma were found, but in this age group they are less important than in allergic diseases [155], [156] (Sintobin, in press).

### 2.7 Biomechanisms of recurrences after surgery

In the same way as concomitant asthma, also the probability of recurrences after total surgical removal might be calculated from biopsies of CRSwNP patients. The claim of completeness is based on the fact that in every polyp an immunological memory is saved in the form of so-called memory cells that maintain the existing immune reaction unless they are surgically removed during an intervention. Surgeries of recurrences in CRSwNP are frequently performed. A follow-up examination over 12 years performed in Ghent, Belgium, revealed recurrences in nearly 80% of the patients requiring at least intensive drug therapy (Gevaert, in press). A total of 36% of the patients underwent at least one revision surgery while the second interventions were performed over all 12 years. Corticosteroid resistant recurrences may even develop 10 years after the first surgical intervention.

In a study performed over a shorter interval of 7 years, we examined the immune profiles that were associated with recurrences [9]. Biopsies of CRSwNP patients who had developed a recurrence within this time were compared to biopsies of patients who had not recurrence within this same period of time. In all specimens the following parameters were measures: Th1, Th2, and Th17 cytokines, IgE, pro-inflammatory cytokines and mediators of eosinophilic (ECP) and neutrophil inflammation (MPO). Patients with recurrences had significantly higher concentrations of total IgE, SE-IgE, ECP, and IL-5 (OR 6.4) at first surgery, whereas IFN-γ was significantly reduced (OR 0.047). The concentrations of IL-17, IL-6, TGF-β1, and IL-1β did not differ between the groups. Asthma and NERD, both Th2 weighted, as clinical parameters were also more frequent in the group of recurrences.

The conclusion can be drawn that the polypous tissue of patients with high risk of recurrences is different from tissue with low risk for recurrence. The immune profile that has to be antagonized can be deducted, and the prognosis of the individual patient can be estimated. Those findings show as well as observations of comorbid asthma how heterogeneous chronic rhinosinusitis is and which great influence is exercised by T helper cells on the disease, their therapy, and prognosis. So we have to move away from the idea that a simple classification into CRSsNP and CRSwNP meets the differentiation of CRS. Beside endoscope and computed tomography we will have to introduce biomarkers into the diagnostic concepts in order to meet the individual requirements in the sense of personalized medicine.

This development has only started. It might be expected that a better knowledge of the pathophysiology does not only contribute to better predict the patients’ prognosis but allows more targeted application of therapeutic options. While a CRS patient with severe fibrosis and moderate neutrophil inflammation may be treated sufficiently long-term with only one rather limited minimally invasive intervention, the treatment of a patient with severe Th2 associated inflammation and significant edema will be directed at removing the whole tissue of the nasal polyposis. This patients would then undergo a much more aggressive and frequent follow-up as well as long-term application of topic glukocorticosteroids, among others, while the CRS patient might have experienced complete healing after three months. For the first time we can offer biomarkers, however, only in the tissue, later certainly also in the serum that also allow to identify those patients already before first surgery who would have to better undergo drug therapy with humanized monoclonal antibodies and other alternatives to be developed instead of surgery.

This perspective will be further developed in the following chapters while only the differentiation in clusters and then their individual treatment modalities will be discussed.

## 3 Cluster analysis of CRS: definition for CRS endotypes

Nowadays, statistical methods allow examining similar disease patterns by analyzing certain clinical or biomedical characteristics. This approach is called cluster analysis that under optimal conditions is independent from the examiner. However, this method is already limited by the fact that a certain choice of characteristics is made for the analysis. The cluster analysis has promoted the differentiation of severe asthma and it could show that asthma has many different phenotypes [7] that have to be treated in differentiated ways.

In the context of a European project called GALEN we have collected the data of several hundreds of CRS patients. From a subgroup of patients (169 CRS and 89 healthy persons) we analyzed the tissue taken from the paranasal sinuses. The analysis encompassed parameters from the field of remodeling, proinflammatory cytokines, T cell cytokines for Th1, Th2, and Th17 lymphocytes, markers of eosinophilic and neutrophil activation as well as IgE and SE-IgE. In this hierarchic cluster analysis, that was uninfluenced apart from that, exclusively tissue markers were included. It was the objective to recognize clusters of CRS based on biological patterns (Tomassen, in press). Only in a second step, the clinical parameters were considered in order to verify the defined clusters regarding their clinical relevance.

The following aspects could be identified: CRS can be divided into 10 clusters that partly have overlapping characteristics. The markers IgE, ECP, IL-5, SE-IgE, and albumin are relevantly associated with each other, the same is true for the markers IL-1, IL-8, and MPO. Thus there is already a separation of neutrophil from eosinophilic inflammations. However, it becomes obvious that in every eosinophilic nasal polyp regularly also neutrophil inflammation is found. In addition, the results show that albumin might be the connective link between eosinophilic inflammation and edema in nasal polyposis while neutrophil inflammation did not correlate with albumin. A total of 6 of 10 clusters was characterized by an eosinophilic and 8 by a neutrophil inflammation. Two of the clusters were only neutrophil and two had no clear inflammatory pattern. This phenomenon is interesting because it shows that CRS may be present even without evident inflammation. Additionally, single clusters revealed increased TNF-α, IFN-γ, or IL-17 concentrations that were usually associated with neutrophil inflammations. Two of those 6 clusters (cluster 9 and 10) with eosinophilic inflammation were SE-IgE positive and had increased IL-5, IgE, and albumin levels in comparison to SE-IgE negative clusters.

**Figure 9:**
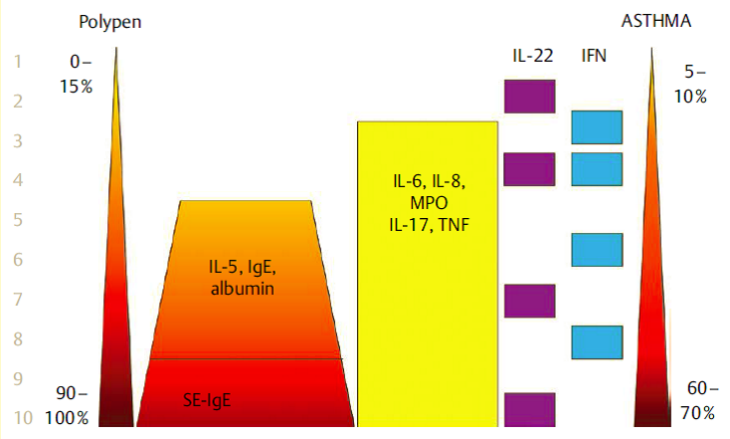
Cluster analysis of CRS: in 8/10 clusters, neutrophil (in yellow) or eosinophilic (in red) inflammation is present. The type with polyps and comorbid asthma (in red) correlates with the eosinophilic inflammation. Both SE-IgE positive clusters who polyposis and comorbid asthma in the majority of the cases.

How can those clusters have a clinical relevance? Figure 9 (Fig. 9) shows the polypous part within each cluster on the left side, while both non-inflammatory clusters were mainly defined as CRSsNP and both IgE positive clusters as CRSwNP according to clinical criteria. In the 6 clusters with eosinophilic inflammation, at least 60% of the cases had been assessed as CRSwNP. On the right side of the figure, the part of comorbid asthma in the patients of the single clusters is depicted. Whereas patients in clusters 1–4 had comorbid asthma in less than 20% of the cases, this percentage in the clusters 9 and 10 is rather high which shows the clinical relevance of the clusters.

Now, the endotypes can be deducted from the cluster analysis that follow certain pathophysiological principles and thus also the same group of therapeutics may be applied, e.g. corticosteroids, but also biologic drugs.

Those endotypes are defined in Table 4 (Tab. 4). It becomes obvious that the eosinophilic endotypes are associated with polyposis and comorbid asthma; however, those characteristics are even clearer in the SE-IgE positive endotype. Parallel, also the risk of recurrence after surgery increases; also in this context the markers of Th2 inflammation as well as the total IgE and SE-IgE that are associated with the incidence of recurrences. Finally, there are only the eosinophilic – mainly the strongly eosinophilic/SE-IgE positive endotype that can be treated by application of innovative therapeutic approaches or biologic drugs that will be discussed later on. So these CRS endotypes have a high clinical relevance and can be used for the prognosis after surgery, prognosis of asthma, and finally prognosis of therapeutic response to targeted biologic drugs. This approach is the base for personalized medicine in the management of CRS.

### 3.1 Special phenotypes

#### 3.1.1 Allergic fungal sinusitis (AFS)

**Figure 10:**
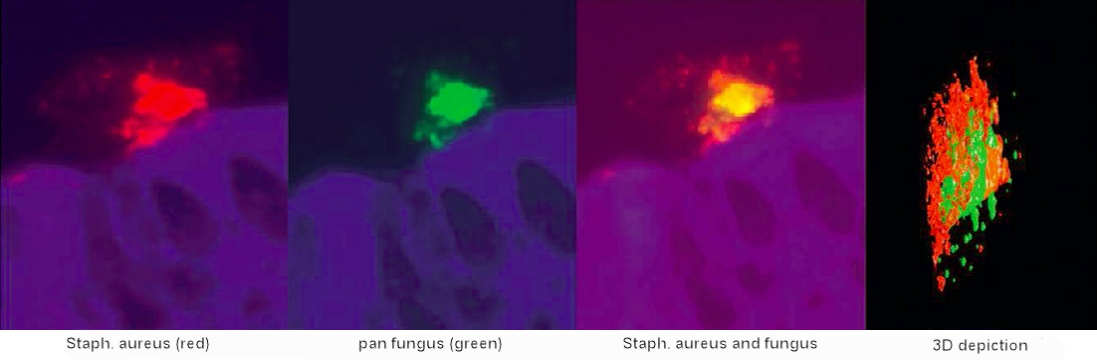
PNA-FISH depiction of *Staph. aureus* and *Aspergillus fumigatus* in patients with AFS

Because of the fact that fungal spores are omnipresent, they permanently enter in the airways by breathing and are deposited on the mucosa. In isolates, *Aspergillus, Penicillium, Cladosporium, Candida, Aureobasidium, *and* Alternaria* are found most frequently. In the airways of healthy people, they rarely become pathogenous, however, sometimes they are able to cause diseases. The non-invasive type of mycotic CRS (fungus ball and allergic fungal sinusitis) are diagnosed more frequently than invasive types [157], [158]. In geographic areas with warmer climate, the prevalence of allergic fungal sinusitis is higher; the presence of fungi such as *Bipolaris spicifera, Curvularia lunata, *or* Aspergillus* species in the paranasal sinuses is typical for the disease [158]. A high total IgE concentration and a relevant eosinophilia in the blood and the mucosal tissue are observed. In contrast, it is unclear how those fungi induce this high IgE concentration because they do not release stimulants for such IgE production, as for example superantigens. More recent data reveal the co-existence of the fungus *Aspergillus fumigatus* with* Staph. aureus* in biofilms in patients coming from Saudi Arabia while *Staph. aureus* is able to trigger the polyclonal IgE production and the severe inflammatory reaction [159]. This observation was confirmed in cases of ABPA (allergic broncho-pulmonary aspergillosis), which is the equivalent of allergic fungal diseases of the paranasal sinuses in the lower airways, by the evidence of SE-IgE antibodies and their correlation with the total IgE (L. Chishimba, Manchester, in cooperation with our lab; publication is prepared). Those observations could lead to a new understanding of AFS and ABPA and thus to a new therapeutic approach of *Staph. aureus* and fungal germs (Figure 10 (Fig. 10)).

#### 3.1.2 Aspirin sensitivity (NERD)

Often an intolerance against aspirin or non-steroidal anti-inflammatory drugs (NSAID) is observed in patients suffering from severe polyposis and asthma. The prevalence of NERD (non-steroidal drug exacerbated respiratory disease) amounts to around 8% in the group of CRSwNP and so it is significantly higher than in controls or CRSsNP. More than 50% of the patients with asthma and aspirin sensitivity also have polyps. NERD often develops as final diagnosis, i.e. after persisting rhinitis in the third decade, followed by asthma, nasal polyposis, finally aspirin intolerance occurs. About half of the patients suffer from severe asthma depending from steroids. Surgery of the paranasal sinuses is associated with a high incidence of recurrences in aspirin-sensitive patients [9], [160].

In diseases of the paranasal sinuses, especially in CRSwNP and NERD, the eicosanoid biosynthesis is modified. The imbalance is mainly characterized by an overexpression of proinflammatory mediators, i.e. of leukotriene, and by a deficit of anti-inflammatory mediators (prostaglandin E2 (PGE2) and lipoxin LXA4). Cysteinyl leukotrienes (cys-LT) are important inductors of airway inflammations [85], [161], [162]. In CRSwNP/NERD the concentrations of those mediators, of enzymes contributing to their biosynthesis (leukotriene synthase, LTC4S, and 5-lipoxagenase, ALOX5) and their receptors are significantly higher in comparison to CRSsNP and control persons. They correlate with the number of activated eosinophils and the concentrations of ECP, IL-5, and IL-5Rα [85]. Furthermore, the ability of tissue of nasal polyps to produce PGE2 and to up-regulate cyclooxygenases (COX)-1, COX-2, and the prostaglandin E receptors EP2 is reduced under proinflammatory conditions [163]. The expression of transcripts of prostaglandin E receptors EP1 and EP3 is down- and the receptors EP2 and EP4 are up-regulated [164]. As the PGE2 concentrations are reduced, the synthesis of leukotrienes (LT) is no longer suppressed so that excessively many eosinophils and high quantities of cys-LT are found [85]. PGE2 could also influence remodeling of nasal tissue induced by fibroblasts [165]. Fibroblasts from nasal polyps produce significantly higher quantities of constitutively expressed COX-2 mRNA than fibroblasts of the inferior turbinates which stimulated vascular dilatation and mucin production [163].

The hematopoietic prostaglandin D synthase (hPGDS) and the microsomal prostaglandin E synthase 1 (m-PGES-1) are both involved in the biosynthesis of PGD2, however, they seem to be regulated otherwise in CRS [166]. The expression pattern of PGD2 receptors such as DP1 (D-prostanoid receptor 1) and CRTH2 (chemoattractant receptor homologous molecule) that is expressed on T helper cells of type 2, is not yet clarified: in CRSwNP the DP1 receptors is found mainly on tissue infiltrating, inflammatory, and constitutive cells while CRTH2 is mostly expressed in inflammatory cells (eosinophils and T cells) [167]. In tissue of nasal polyps, the de novo synthesis of PGD2 after stimulation via IgE is significantly up-regulated [113], and this release promotes the release the migration of Th2 cells via a CRTH2-dependent mechanism [86].

As can be read in recent studies, the progression of inflammation in NERD airway diseases is associated with a comparably reduced lipoxin synthesis while the mRNA expression of ALOX15 and the consecutive LXA4 concentrations in CRSwNP patients is up-regulated [85]. ALOX15 is increased in the epithelium and subepithelium and correlates positively with the number of activated eosinophils infiltrating the mucosa of the nasal polyps; NERD patients have a reduced basal synthesis and reduced ability to synthesize LXA4 under proinflammatory circumstances [85], [168].

## 4 Pharmaco-therapy of chronic rhinosinusitis

The options that are currently recommended for therapy of chronic rhinosinusitis (CRS) are summarized in international and national guidelines. Most wide-spread are the EPOS guidelines that have been established by an international team of experts (including myself) according to criteria of evidence-based medicine [1]. Among those treatment recommendations, topical and systemic glukocorticosteroids, antibiotics as long-term therapy or in cases of exacerbations as well as irrigations with saline solution are found. If those options do not lead to successful treatment or control of the symptoms, generally surgery is discussed. Postoperatively the same pharmacotherapeutics are continued or applied. The recommendations of the EPOS guidelines are summarized in Table 5 (Tab. 5) and Table 6 (Tab. 6) for CRS without nasal polyposis (CRSsNP) and CRS with nasal polyposis (CRSwNP). They can be completely downloaded via internet (http://www.rhinologyjournal.com).

### 4.1 Chronic rhinosinusitis without nasal polyps (CRSsNP)

Beside topical corticosteroids that are less effective in CRSsNP before surgery because of the eosinophilic character and the poor accessibility of the paranasal sinuses compared to CRSwNP, especially macrolides can be discussed as low-dose long-term therapeutics (>12 weeks) in CRSsNP. Even if the EPOS guidelines actually recommend macrolides unless IgE is not elevated in the serum, an extensive European study with azithromycin was negative [169]. However, there was criticism about the patient selection (CRSsNP and CRSwNP) and the missing consideration of serum IgE. A. Cervin who was very committed to macrolide therapy summarized 30 years of experience with microlides and many open studies in a recent review article that only two randomized controlled studies had been performed that contradict regarding their results. In one study that treated only CRSsNP patients with roxithromycin versus placebo a positive clinical effect was observed after 12 weeks of treatment. However, recently major concern have arisen in the context of macrolides as they might cause cardiac arrhythmia [170]. In patients with risk factors such as a prolonged QT intervals, bradycardia, hypokalemia, hypomagnesaemia etc. a long-term application of macrolides cannot be recommended. Macrolides may also interact with the cytochrome P450 system, cause ototoxicity, and finally lead to development of resistances to macrolide antibiotics in case of broad application [171].

### 4.2 Chronic rhinosinusitis with nasal polyps (CRSwNP)

The application of topical corticosteroids is not disputed in nasal polyps, as initial but also as postoperative therapy. A dosage of 2x daily has to be observed which can be reduced to 1x daily if reasonable when the disease is controlled (for example no recurrence or no exacerbation over at least one year after surgery). Because of the necessity of high dosage over several years, substances with a low bioavailability should be preferred; according recommendations are included in a therapeutic index [172]. In particular after surgery topical corticosteroid as drops are recommended. They are applied in a position with the head bent over or in the so-called Mecca position in order to distribute optimally in the ethmoid region and at the skull base. Recurrent polyps would preferably develop in these regions.

The effectiveness of doxycycline (100 mg/d) has been proven in a study of patients suffering from nasal polyposis over 20 days [123]. In this study, the effect of oral corticosteroid therapy on symptoms, objective clinical as well as biochemical parameters was compared to the one of doxycycline. A placebo group was also created. In this double blind randomized placebo-controlled study 47 patients with bilateral nasal polyposis either underwent therapy with methyl-prednisolone in decreasing dosage (from 32 to 16 to 8 mg every 5 days) or doxycycline or placebo therapy (200 mg on the first day). The patients were monitored over 12 weeks by means of peak flow measurements, endoscopy, symptom scores, and biomarkers in nasal secretion and peripheral blood. It became obvious that methyl-prednisolone and doxycycline caused a significant reduction of the size of the polyps compared to placebo. The effect of methyl-prednisolone was maximal after 2 weeks but before the end of therapy it already decreased and in total it was only significantly different of placebo over 8 weeks. The effect of doxycycline was less relevant but significant over the 12 weeks of evaluation. Methyl-prednisolone significantly suppressed the mediators ECP, IL-5, and IgE in nasal secretion while doxycycline reduced the concentrations of MPO, ECP, and MMP-9.

We drew the conclusion of this study that an oral corticosteroid application has only a relatively short effect on CRSwNP and that the side effects – especially in cases of repeated application – are outweighed. In practice we avoid oral corticosteroids as far as possible, in particular directly preoperatively because the blood eosinophils show a clear rebound effect after treatment. A high number of circulating eosinophils a few weeks after surgery must be considered as prognostically unfavorable.

Because of its positive effect on bacterial colonization including *Staph. aureus* and biofilm formation as well as because of reduction of MMPs (which contribute to the development of edema in nasal polyposis) and IgE, doxycycline is applied as long-term therapy for several weeks, especially in the postoperative phase in order to reduce edema and scar formation. The recommendations of the ENT Department of Ghent are summarized in Table 7 (Tab. 7).

It is obvious that those therapeutic principles are insufficient in some patients; even if a temporary improvement of the complaints can be achieved it cannot be maintained over a longer period of time. Especially in patients with CRSwNP pharmacotherapy is often not sufficient so that surgery becomes necessary. Recurrences occur frequently even after surgery so that some of the patients have to undergo surgery several times in their lives. A follow-up evaluation performed at the University of Ghent in 42 patients older than 12 years estimated the recurrence rate over 12 years with more than 75% while a total of 36% of the patients had to undergo at least a second intervention (Gevaert, in press). Another study from Ghent had identified biomarkers previously that in the case of necessary second intervention had already been increased at the time of first surgery in comparison to patients who did not need second surgery (here over 7 years): IgE, SE-IgE, ECP, and IL-5 [9]. Those markers can all be classified into the Th2 pattern. A high interferon level (IFN-γ, Th1 cytokine) could be measured in patients who did not require revision surgery. This suggests perspectives for a prognosis based on the biomarkers measured at the occasion of the initial intervention. Furthermore, it seems to be reasonable to apply therapeutics that are effective for eosinophilic inflammations, in particular glucocorticosteroids, in this patient population rather long and in higher doses. Additionally accompanying measures against *Staph. aureus* increase the control of the inflammation, for example by application of doxycycline or mupirocin.

For patients who underwent complete resection with the objective of the inflammatory tissue, who were treated postoperatively with the above-mentioned therapeutics, and who nonetheless had developed recurrences indicating surgery, the current treatment options are apparently insufficient. Innovative approaches, actually in particular so-called biologics, have to be considered. Regarding this development, we are currently only at the beginning. Currently only one humanized antibody is at our disposition limited to patients with the indication of severe allergic asthma that cannot be treated otherwise. In the near future, however, it might become obvious that those patients can already be identified before initial surgery by determining biomarkers and – as surgery will most probably not lead to the control of the disease – alternative therapy can be initiated immediately. Those reflections have to be made also with regard to the background of cost and benefit/risk analyses.

#### 4.2.1 Specific reflections on allergic fungal sinusitis (AFS)

Treatment starts with the complete resection of fungal masses and colonized *Staphylococcus* as well as the polypous mucosa from the paranasal sinuses. Attention must be paid to complete removal and intensive postoperative care like in other eosinophilic polyps. Just as eosinophilic nasal polyps, the disease is also characterized by a Th2 pattern; IL-5 is much increased such as IgE, there are numerous secreting plasma cells and activated T cell that apparently dispose of a memory for specific antigens. The postoperative application of oral and topical corticosteroids supports the reduction and termination of the immune response.

#### 4.2.2 Specific reflections on aspirin sensitivity (NERD)

Also nasal polyps in aspirin sensitivity or NSAID exacerbated respiratory diseases show a clear Th2 pattern where the markers of eosinophilic inflammation (IL-5, ECP etc.) and also the tissue IgE are very high [173]. SE-IgE is generally positive in the tissue and in cases of asthma also in the serum. In comparison to normal polyposis with asthma, a deficient production of LXA4 is obvious which has anti-inflammatory activity. Apart from that, surgery as well as conservative therapy of CRSwNP in NERD follow the same principles as described above (treatment scheme of Ghent, Table 7 (Tab. 7)). Also the application of omalizumab is not excluded.

Advocates of an adaptive deactivation must accept the reply that only few centers perform provocation with aspirin or lysine aspirin which is a precondition of adaptive deactivation; that a series of contraindications of provocation must be observed [174]; that the benefit of this therapy, especially postoperatively, is not proven; and that there are very contradictory ideas on effective but well tolerable dosage of aspirin [174]. 

## 5 Biologics for the treatment of asthma and chronic rhinosinusitis

It is clear that the pharmaceutical industry has experienced an unparalleled development of pharmacologically active peptide- and protein-based treatment approaches for therapy of different diseases of the mucosa of the airways and the gastrointestinal tract as well as systemic diseases (for example Crohn’s disease, multiple sclerosis, rheumatoid arthritis, just to mention some of them) during the last years. The development of such biological substances requires profound knowledge of specific molecules of which the binding or “deactivation” has a relevant therapeutic effect. Only then it makes sense to develop antibodies and to humanize them secondarily, i.e. to approximate them to the human antibody product as near as possible. This process is incomparably expensive in relation to the development of classic drugs. Currently about 200 biologics are used for patients, further 400 are tested in studies. Among the indications, also severe asthma is found that is very similar to chronic rhinosinusitis regarding its immune mechanisms, especially to the Th2 pattern of CRSwNP.

Different innovative biologics are tested in clinical studies for severe asthma and up to now finally one has been approved. Omalizumab is applied today in a relatively specific small group of patients with severe asthma, which cannot be treated otherwise, under strict conditions. One of the preconditions is that standard therapy with corticosteroids in high doses is not sufficient, that perennial asthma is present, and that a lung specialist confirms the indication of omalizumab. Omalizumab is a humanized monoclonal antibody that is targeted against fee IgE. It was primarily developed as antiallergic agent [175]. Meanwhile studies show that omalizumab is also effective without the presence of allergy in cases of severe asthma (the so-called intrinsic asthma) while those patients also have increased IgE serum levels (see above) that are not targeted against inhalative allergens. Insofar the limitations of the authorities regarding the application of omalizumab are already obsolete.

Also for chronic rhinosinusitis the single state-of-the-art studies (DBRPC) with biologics have been performed, up to now always in Ghent (Coordinating investigator: Claus Bachert); reports on the experiences with omalizumab have also been made by other departments. Among the tested humanized antibodies, also anti-IgE (omalizumab), anti-IL-5 (reslizumab and mepolizumab) as well as anti-IL-4/13 (dupilumab) can be found. The target molecules were targeted against Th2 associated mediators each. The studies reveal that the mentioned biologics are therapeutically effective in 60-80% of the patients. In the future, biomarkers shall help to increase this response rate. Without any doubt, the studies can also be valued as proof-of-concept for the possibility of targeted therapy of Th2 associated mediators in cases of eosinophilic chronic rhinosinusitis with nasal polyps with or without concomitant asthma.

Table 8 (Tab. 8) and Table 9 (Tab. 9) give an overview of possible target molecules for the treatment of Th2 associated respiratory diseases (CRSwNP and asthma) according to the literature available today.

### 5.1 Anti-IgE (Omalizumab)

The discovery of high concentrations of IgE in nasal polyps [115], [176] and the understanding that those IgE antibodies are polyclonal and still functional [110], has justified a therapeutic attempt with omalizumab, a humanized monoclonal antibody against free IgE. After positive reports on single cases [177], the first smaller DBPCR study with omalizumab in chronic rhinosinusitis, however, had negative results; the reason was probably that patient selection was made too inhomogeneously because also patients without nasal polyposis were included where IgE does not play a major role [178].

The effect of omalizumab is based on binding to the Cε3 region of the Fc fragment of the IgE molecule; the same structure also binds to the alpha chain of the highly affine receptor (FcεR1) on mast cells and basophils. The binding of omalizumab thus inhibits docking of IgE to the highly affine receptor, but binds in complexes that circulate in the blood for a long time and are finally degradated. Hence, omalizumab does not only bind free IgE in the serum but disarms mast cells and basophils in the tissue and blood that do no longer degranulate in cases of contact with the allergen or do no longer induce anaphylaxis [179]. As consequence, the expression of FcεR1 on these cells is reduced and the consecutive effects including eosinophilia are less important. At the same time the binding of IgE to the lowly affine receptor FcεRII is inhibited which reduces the IgE induced antigen presentation by dendritic cells. In this way the allergic reaction is again significantly attenuated.

Today, omalizumab is mainly applied as successful therapy in severe allergic asthma if asthma cannot be controlled by conventional asthma therapy and the patient is at risk because of exacerbations of his disease. Frequently, there is the additional requirement that a perennial allergen is proven, the meaningfulness of this claim must be questioned. The dosage of omalizumab should lead to a relevant reduction of the free IgE concentration in the blood, at the same time also IgE in the serum decreases. The dosage is calculated based on the total IgE in the blood (max. 1,500 kU/l) as well as the body weight of the patient. In cases of severe asthma, omalizumab leads to a significant reduction of the exacerbations of the disease and consecutively to a reduction of hospitalization (as well as the associated costs). Furthermore, it reduces the number and the impact of viral diseases especially in children [137]. Currently an even more effective anti-IgE molecule is being approved.

Despite the previous work on local production of IgE in nasal polyps and their functionality it was not clear if omalizumab reached the poorly vascularized tissue of nasal polyps in sufficient concentration and could develop its therapeutic effect in the sense of reduction of the nasal polyposis after binding of IgE. Additionally, about half of the patients are not atopic so that again the question of the importance of the IgE antibodies was asked. In those patients, asthma was often not allergic but classified as late-onset asthma. However, we had already explained previously that an increased IgE or SE-IgE in tissue of nasal polyps is found in 30–40% of the patients with CRSwNP, significantly increasing the risk of asthma [152]; furthermore we had also proven the functionality of local IgE antibodies [110]. The effectiveness of omalizumab would be another proof of the functionality of local IgE production in the upper and lower airways and would support the role of a non-atopic IgE synthesis induced by *Staph. aureus*.

The clinical effectiveness of omalizumab applied in the usual doses for severe asthma was examined in patients with severe polyposis and concomitant asthma (light to moderate) [180]. Omalizumab significantly reduced the score of nasal polyps (primary parameter) already after 8 weeks and after 16 weeks further reduction of the nasal polyps was found of a total of 2.6 score points; this corresponds to the effect of a therapy of 3 weeks with oral glucocorticosteroids (32 mg of methyl-prednisolone) and thus it is clinically relevant. Further, omalizumab significantly reduced the Lund-Mackay score, which measures the involvement of the paranasal sinuses in the disease, as well as the symptoms of the upper and lower airways including nasal obstruction, rhinorrhea, and smelling disorders, wheezing, and shortness of breath. Also quality of life parameters significantly improved with omalizumab in comparison to placebo, including the asthma quality of life (AQLQ). So it could be confirmed for the first time that the reduction of local IgE in selected CRSwNP patients was a worthwhile therapeutic objective.

Half of the patients treated with omalizumab were positive against inhalative allergens in the skin test and thus characterized as allergic. The others had no allergies. Although the number of patients in this study was limited and further studies will be necessary to clarify the effectiveness especially of non-allergic patients, a comparative analysis revealed a better response of non-allergic than allergic patients, especially in cases of asthma symptoms. Meanwhile, there are also reports on experiences with non-allergic asthma that confirm our results [181].

**Figure 11:**
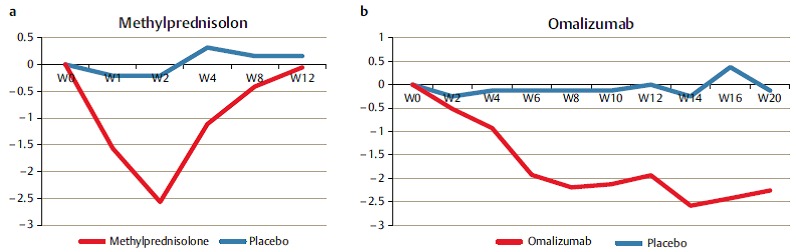
Changes of the nasal polyp score after a) methyl-prednisolone for 3 weeks (32/16/8 mg) and b) omalizumab (recommended dose for asthma)

In this way, omalizumab demonstrated its clinical effectiveness in patients with nasal polyps and concomitant asthma over several weeks and it was well tolerated. Known risks such as anaphylactic reactions occurring in rare cases must be observed. Experiences in the clinic with patients who were treated primarily because of severe asthma and who had CRSwNP at the same time, confirmed the positive effects on the growth of the nasal polyps [182]. Nowadays we successfully apply omalizumab in patients with corticosteroid resistant recurrences of polyposis after at least one correctly performed surgery of the paranasal sinuses (in the sense of complete resection of the polyps) and concomitant (mostly not severe) asthma complaints. So this situation is outside asthma indication and we justify the application with the failure of conventional pharmacotherapy and surgery. Of course, the currently (still) high costs of the treatment have to be included in the indication (Figure 11 (Fig. 11)).

### 5.2 Anti-IL5 (Reslizumab, Mepolizumab)

About 80–85% of the nasal polyps in Europe, but only 10–50% of the polyps in Asia, have a Th2 inflammation pattern with prominent eosinophilia, expression of interleukin-5, and typical chemokines as well as activation markers for eosinophilic granulocytes. As IL-5 plays a key role in chemotaxis, differentiation, activation, and the survival of eosinophils and as those cells represent such a prominent characteristic in the polyps, the antagonism of IL-5 could offer a therapeutic chance. However, it was unclear if anti-IL-5 really reached the polyps and led to a reduction of eosinophils with diminution of the nasal polyps because polypous formations are also possible without eosinophilia and the association of eosinophils with edema is unclear.

In 1997, IL-5 was identified as key cytokine in nasal polyposis [100]; among a series of cytokines, IL-5 was most clearly associated with eosinophilia in nasal polyps and could be proven immunohistochemically also in eosinophils so that the cells themselves could be identified as source of the cytokine. In the same year we could also show in an ex-vitro model that anti-IL-5 antibodies, but not anti-IL-3 or anti-GM-CSF lead to cellular death (apoptosis) of the eosinophils [120]. Later, it could also be revealed that the regulation of IL-5 receptors plays a significant role in the localization of the cells in nasal polyps [183]. A first pilot study with reslizumab, a humanized anti-IL-5 antibody, showed a significant reduction of the size of the polyps in about 50% of the non-selected patients after only one intravenous injection. A high IL-5 level in nasal secretion seemed to predict the therapeutic response and also clearly decreased under verum therapy [184]. Hence, the principle of IL-5 antagonism was established in eosinophilic nasal polyps.

In a second study, this time with 2 IV injections of 750 mg of mepolizumab, of 30 patients with severe nasal polyposis (Davos 3-4 or recurrence after surgery) even with topical corticosteroids the results were mostly confirmed [101]. After 8 weeks, a significant reduction of the polyp score of at least one point was found in 60% of the patients treated with mepolizumab as well as a significant reduction of the CT score. Those patients who had a reduction of the polyp score of at least one point (“responders”) observed a remaining effect over nearly one year. So mepolizumab had a clinically relevant and in selected patients also long-lasting effect on nasal polyposis that did no longer respond to topical corticosteroids and thus would have had to undergo surgery. Like omalizumab, the quality of the effectiveness can certainly be considered as new dimension of therapeutic possibilities in nasal polyposis. Again, mepolizumab was well tolerated. Another long-term study with mepolizumab (principal investigator: Claus Bachert) has just been closed. The results are expected to be present in 2015. In the same year, also the approval of mepolizumab for asthma can be expected. However, currently it is still unclear if the drug will be approved for nasal polyps.

### 5.3 Anti-IL-4/-13 (Dupilumab)

As severe persisting respiratory diseases like CRSwNP and asthma must be classified as Th2 inflammations, innovative therapeutic approaches focus on Th2-associated cytokines. Beside interleukin-5, also IL-4 and IL-13 must be mentioned. Both cytokines act via two different receptors that partly overlap in their functions and they both contain the alpha sub-unit of the IL-4 receptor. The type 1 receptor that can only activated by IL-4 is mainly expressed on lymphocytes and regulates the differentiation of those cells. The type 2 receptor that is activated by IL-4 and IL-13 is expressed by a multitude of cells and has different functions, among them the production of the mucous secretion that is typical for nasal polyps. Dupilumab (SAR231893/REGN668) is a humanized antibody against the alpha sub-unit of the IL-4 receptor and can inhibit the effect of both cytokines, IL-4 and IL-13.

An investigation performed in the USA on severe asthma, the application of dupilumab could reduce the number of asthma exacerbations by 87% in comparison to placebo [185]. In this study, the patients were deprived of their asthma medication which led to a significant increase of exacerbations in the placebo group but not in the verum group. The pulmonary function in the verum group improved significantly. Furthermore it could be observed that different biomarkers of eosinophilic inflammation (FeNO, serum-IgE as well as the chemokines eotaxin-3 and TARC) were significantly reduced in the serum which confirms the biological activity of dupilumab. The clinical effect size of dupilumab seemed to be higher as the one of other biologics. As IL-4 and IL-13 also play a role in the pathogenesis of nasal polyps, we have performed a DBPCR study on the effectiveness and security of dupilumab in patients with CRSwNP with at least 5/8 score points in the polyp score with or without concomitant asthma. A total of 60 patients with bilateral nasal polyposis despite double dose of topical corticosteroids (mometasone furoate 2x daily) were observed for 4 weeks with topical corticosteroids and then they were treated either with 300 mg of subcutaneous dupilumab per week or placebo for additional 16 weeks. In the verum group the polyp score significantly decreased of 1.9 points compared to placebo (–0.3); also the CT score according to Lund and Mackay was significantly reduced (–8.8). The volume of the polyps in the maxillary sinuses was reduced by 32%, the SNOT-22 and the UPSIT smelling test revealed a significant effect compared to placebo. More than half of the patients responded with a reduction of the polyp score of at least 2 points (nearly similar to the effectiveness of 3 weeks of oral application of corticosteroids). The symptoms like nasal obstruction, rhinorrhea, and the subjective as well as objective smelling significantly improved in the verum group as also the nasal peak flow (PNIF, improvement of 60%).

Patients with asthma achieved a significantly improved pulmonary function (FEV_1_) and asthma control test (ALQ-5) in comparison to placebo. Patients with asthma also had a better reduction of the score of polyps compared to the total group (–2.4 score points in comparison to placebo). 56% of the patients had a reduction of the score of nasal polyps of more than 2 points.

Biomarkers of eonsinophilic inflammation were also significantly reduced, among them IgE, TARC, and eotaxin-3; in contrast to anti-IL-5, however, the absolute number of eosinophils in the blood died not decrease. The action profile of both biologics can be well differentiated while the clinical effectiveness of dupilumab seems to be superior to mepolizumab with comparable tolerability.

The results of this study are consistent with previous investigations of severe asthma and atopical dermatitis and they impressively confirm the potential of dupilumab to inhibit each type of Th2 induced inflammation and to reduce clinical complaints. For nasal polyps we could confirm this fact by subjective as well as objective parameters as additional effect being superior to basic therapy with topical corticosteroids. Also in this context the effect size is similar to the one of oral corticosteroids. Comparable to omalizumab, the patients with asthma also benefit from the significant improvement of their complaints regarding the lower airways.

### 5.4 Biomarkers

It is obvious that future ENT specialists will need other diagnostic tools beside endoscopes and CT scans allowing them to identify the endotype of each disease. Such surrogate markers could be determined most simply in the serum of the patients, as for example the absolute number of eosinophils in the blood, ECP as marker of activation of those cells, the total IgE as parameter of local IgE synthesis, IL-5Ra instead of IL-5 etc. Further possibilities are probably also chemokines such as TARC, PARC, eotaxin-3, or periostin. However, those markers are certainly increased in the tissue but – in contrast to asthma where the lung has a bigger production surface – the markers in the serum are often below the detection limit which seriously impairs the sensitivity of the test. 

A primary objective would be to identify the Th2 type in the tissue. Probably even specific statements can be given on the involvement of single cytokines such as IL-4 vs. IL-5. The more successfully the individual endotype can be defined, the more precise is the statement about the prognosis (recurrence, asthma) and a personalized therapy can be started with prediction of the therapeutic effect of the according biologic. It must not be forgotten to mention that also other T helper cell reactions might be interesting for therapeutic interventions such as for example Th17, Tfh, Th22, and others. Currently large European cohort studies are performed on the question of serum markers.

### 5.5 Future approaches

There are a series of further therapeutic objectives as for example the antagonization of single cytokines of Th2 inflammation or their receptors like the IL-5 receptor alpha, TSLP. But also the deactivation of certain cells, especially of IgE producing B cells via a particular structure of the B cell binding molecule, M1 Prime, may even be a superordinate aim of research.

Although the development of those biologics is very promising, there are still some problems like for example the high costs of production and development of those substances, the necessity of systemic application, and the long-term effects that sometimes cannot be excluded. For instance, the question was discussed if omalizumab might increase the risk of malignant diseases. This suspicion could recently be disproved after many years of application [183].

It would be ideal to prefer local applications in the airways that minimizes the systemic risks, that achieves the therapeutic goal with small quantities, and that is consequently cheaper. As representatives of a series of such local approaches, two different approaches will be discussed here that have already been tested in humans and that provided the evidence of clinical effectiveness in mucosal surfaces.

#### 5.5.1 Gene silencing, GATA3 DNA-zymes

The Th2 endotype is characterized by the predominance of activated Th2 cells that synthesize and release the cytokines IL-4, IL-5, and IL-13. The expression and production of those cytokines is controlled by the transcription factor GATA-3 that is relevant for the differentiation and activation of Th2 lymphocytes and is considered as “master switch” for all Th2 associated diseases. GATA-3 is overexpressed in nasal polyps, in asthma, and in atopic eczema. By reducing the released cytokines, a deactivation of the “master switch” would block also consecutive pathomechanisms such as for example the IgE production.

An innovative approach could thus deactivate all Th2 cytokines via a blockage of the transcription factor GATA-3 and also the associated phenomena of IgE production and tissue eosinophilia [186]. As GATA-3 is expressed intracellularly, so-called “antisense” or “silencing” techniques could be applied; molecules would have to be used the can penetrate the target cells (Th2). This approach was successful for GATA-3 via the development of a GATA-3 DNA-zyme in the sense that the DNA molecule docks to the GATA-3 RNA and quasi “cuts it up” by means of a catalytic enzyme. The DNA-zyme hdg40 is very stable and highly specific, at the same time, however, it is very active and easy to apply via inhalation or spray to the mucosa. In our laboratory we could show that GATA-3 DNA-zyme is absorbed without further transportation support in the T cells and significantly reduces the expression of GATA-3 RNA as well as IL-5 protein [187]. Applied in an asthma model in humans, hgd40 led to a significant reduction of the allergic immediate and late phases as well as the markers associated with a Th2 reaction. Thus it had a high potential in the treatment of Th2 associated diseases including CRSwNP and asthma, but also allergic rhinitis.

#### 5.5.2 Genetically modified Lactococcus lactis (GM-LL)

Instead of complex antibodies that have to be produced and humanized in expensive processes in order to be applicable systemically, it is also possible today to choose approaches where proteins or their derivatives, for example nano-proteins, are produced direction on the mucosa by apathogenous bacteria. ActoGeniX, a spin-off of the University of Ghent, Belgium, has established the technology for development and production of genetically modified *L. lacti* and shown their pre-clinical and clinical applicability for a series of diseases, among them also allergic diseases. Some advantages are the local application, the possibility of producing every protein by GM-LL, and finally the possibility to have produced several therapeutically effective proteins by one single germ. This approach is currently investigated in pre-clinical studies.

#### 5.5.3 Final remarks on biologics

It became obvious that the therapeutic approaches pursued up to now, as they are also summarized in the new EPOS guidelines, do not lead to long-term control of the symptoms or healing in all patients with their basic pillars of corticosteroids, antibiotics, and surgery. It is especially the group of Th2 associated diseases that is often accompanied by recurrences and comorbid asthma. The increased expression of the transcription factor GATA-3, the increased production of IL-4, IL-5 and IL-13 as well as an increased local production of IgE are characteristic for this immunologic situation and at the same time they indicate innovative objectives of intervention.

Biologics have a new opened dimension of effectiveness in comparison to oral corticosteroids; the response occurs more slowly but there effect is longer-lasting and they can be applied for several years without accumulation or side effects with comparable or even better response. Permanent application of oral corticosteroids would undoubtedly lead to health detriment that cannot be accepted. Currently, in particular the costs are an argument against the application of biologics.

It is certain that biologics currently will not replace the surgical possibilities where surgery leads to permanent success, for example in the therapy of CRSsNP. On the other hand, there are patients who suffer from severe Th2 inflammation with high probability of recurrence and the necessity of repeated interventions and with comorbid asthma where surgery cannot have a permanent effect and so in those cases an alternative therapy should be started. For those patients, biologics are a valuable alternative to repeated surgical revision. They are able to control the symptoms of the disease of the upper as well as the lower airways which according to our experience is often associated with a significantly increased quality of life.

We as ENT specialists, however, have to improve our knowledge of immunology in order to have sufficient knowhow in diagnostics and finally therapy with innovative drugs. We have to integrate the principle of endotyping at least in patients with severe respiratory diseases in our daily practice, refine the diagnostics by means of biomarkers, and understand the indications and side effects of biologics with the background of pathophysiology. Only then we will be in a position to offer our patients the possibilities of modern innovative treatment.

## Notes

### Conflict of interests

As principal investigator, Claus Bachert has contributed to studies with topical and oral corticosteroids, antibiotics, and biologics including omalizumab, reslizumab, mepolizumab, and dupilumab.

### Funding

Claus Bachert’s projects have been supported by the Fund for Scientific Research Flanders (FWO-Vlaanderen: Mandat 1841713N and Projecte 3G.0489.08, Go39412N, G.0642.10N, G067512N), the University of Ghent BOF14/GOA/019 and 01J01113, the Interuniversity Attraction Poles Program (IUAP) – Belgian State – Belgian Science Policy P6/35 and P7/30, the European Commission’s 6th and 7th Framework programs FP-6/7 GALEN and PREDICTA.

## Figures and Tables

**Table 1:**
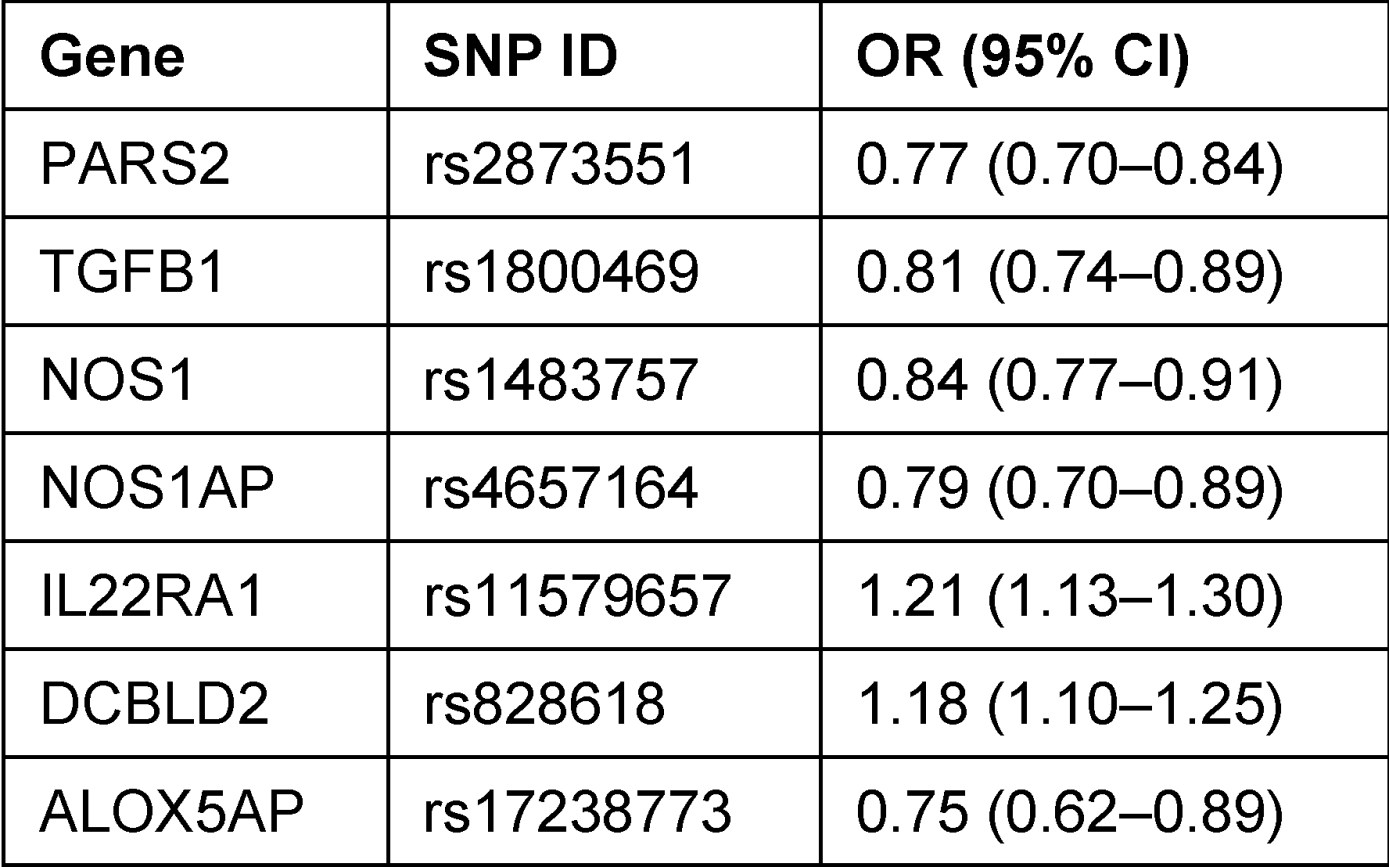
List of SNPs that were associated with CRS in former publications and that could be replicated in our investigations. The more those associations are confirmed in other cohort, the more probable is their significance [12]. ALOX6AB, arachidonate 5-lipoxygenase-activating protein; DCBLD2, discoidin, CUB and LCCL domain containing 2; IL22RA1, interleukin 22 receptor, alpha 1; NOS1, nitric oxide synthase 1; NOS1AP, nitric oxide synthase 1 adaptor protein; TGFB1, transforming growth factor B1.

**Table 2:**
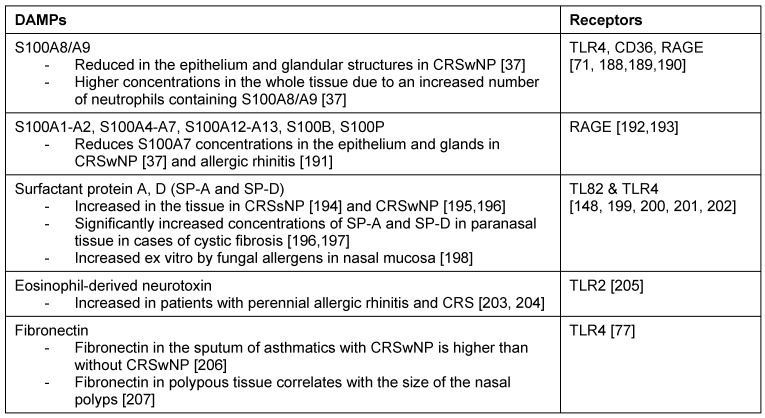
Overview of the expression of damage-associated molecular pattern (DAMPs) and their receptors in CRS and other diseases of the upper airways

**Table 3:**
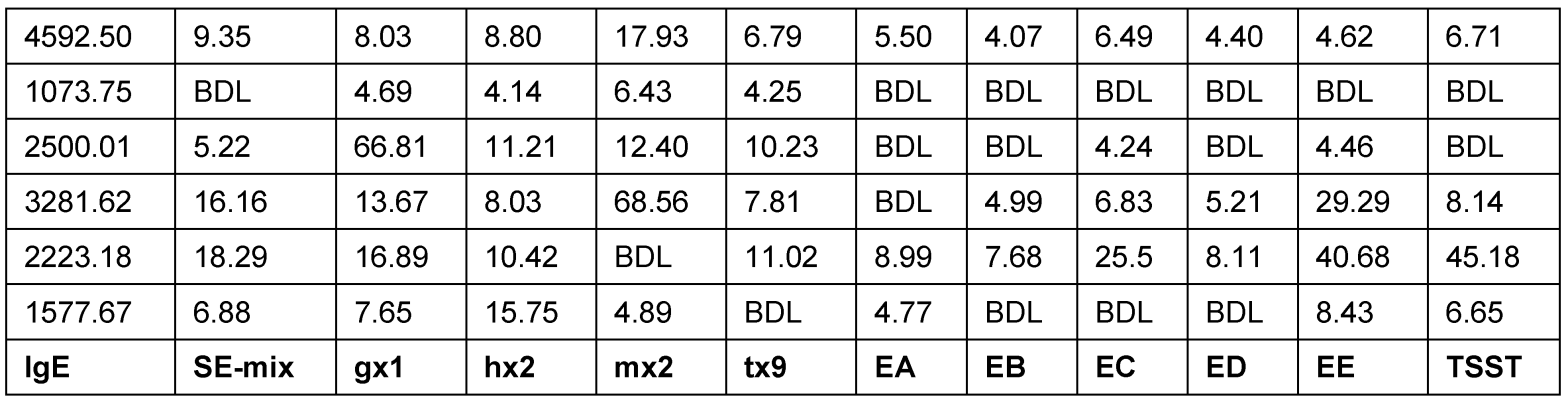
Polyclonal IgE in the tissue of CRSwNP [209]: High total IgE concentrations and numerous IgE specificities characterize the local IgE in nasal polyps. Specific IgE against: gx1 grass pollen mix, hx2 house dust mites mix; mx2 fungal allergen mix; tx9 tree pollen mix; superantigens SEA – TSST

**Table 4:**
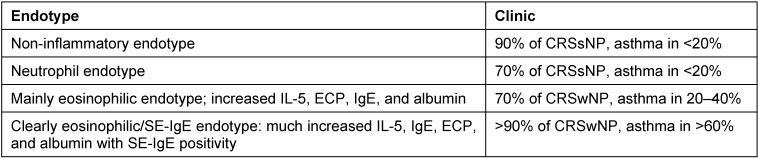
Endotypes of CRS with clinical findings

**Table 5:**
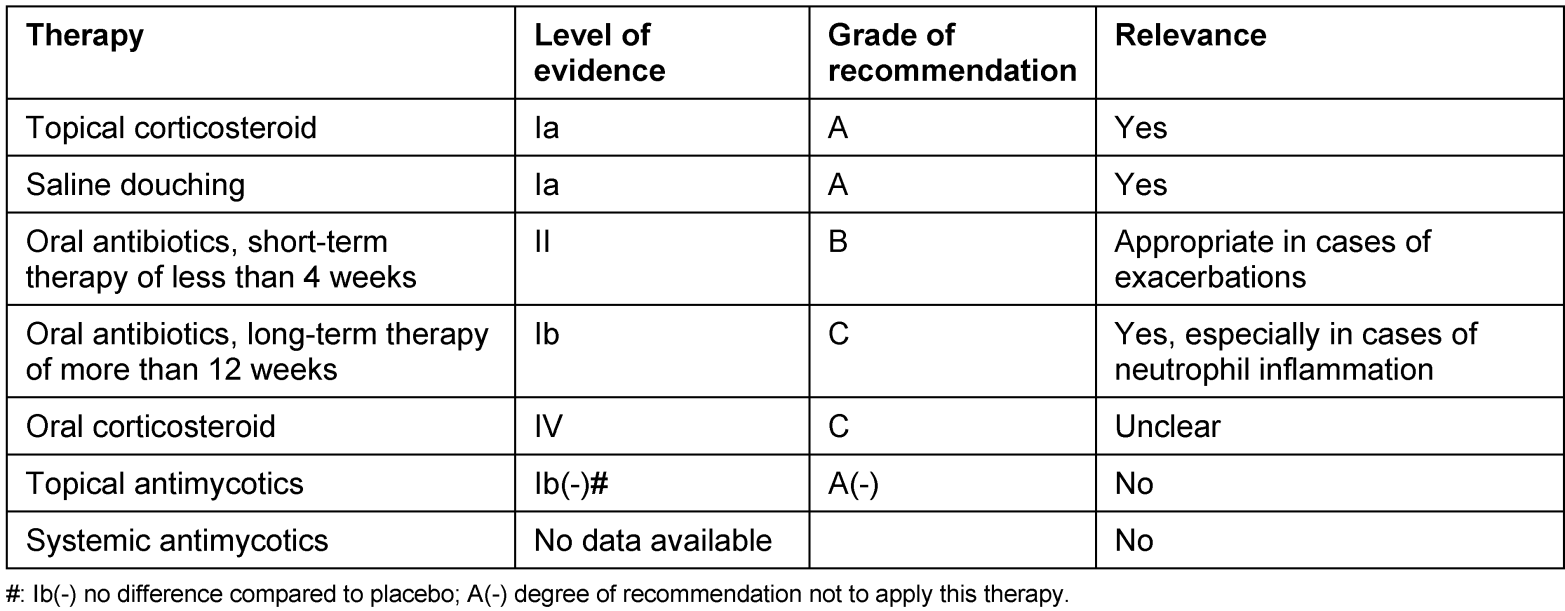
Evidence and recommendations of therapy in CRSsNP for adults (modified according to [1])

**Table 6:**
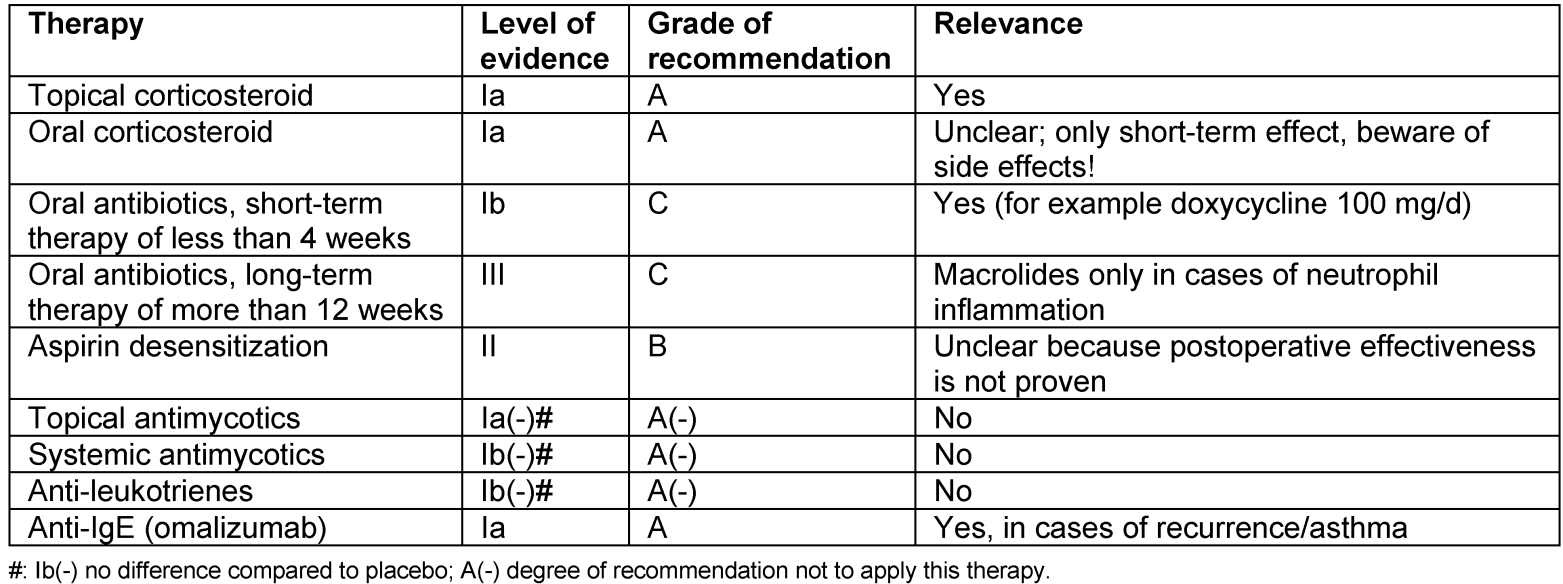
Evidence and recommendations of therapy in CRSwNP for adults (modified according to [1])

**Table 7:**
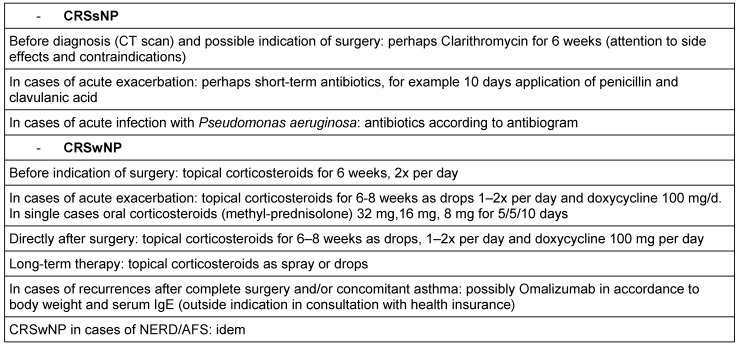
Treatment scheme for CRSsNP and CRSwNP from Ghent

**Table 8:**
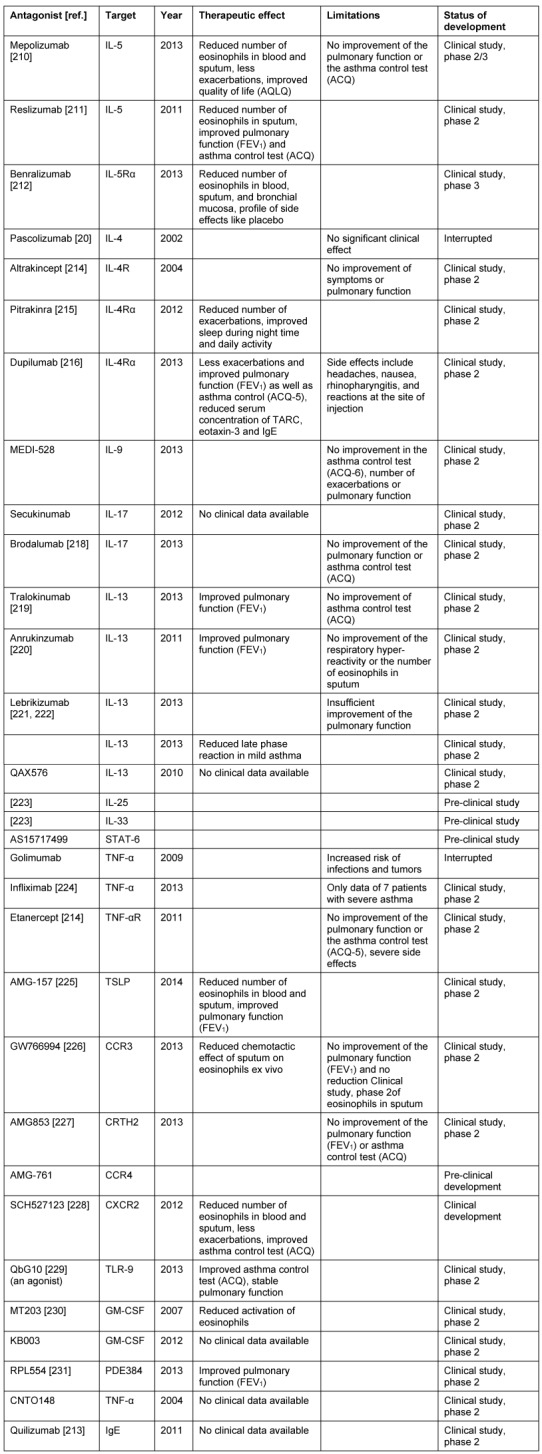
Biologics and other innovative therapeutic concepts for treatment of asthma

**Table 9:**
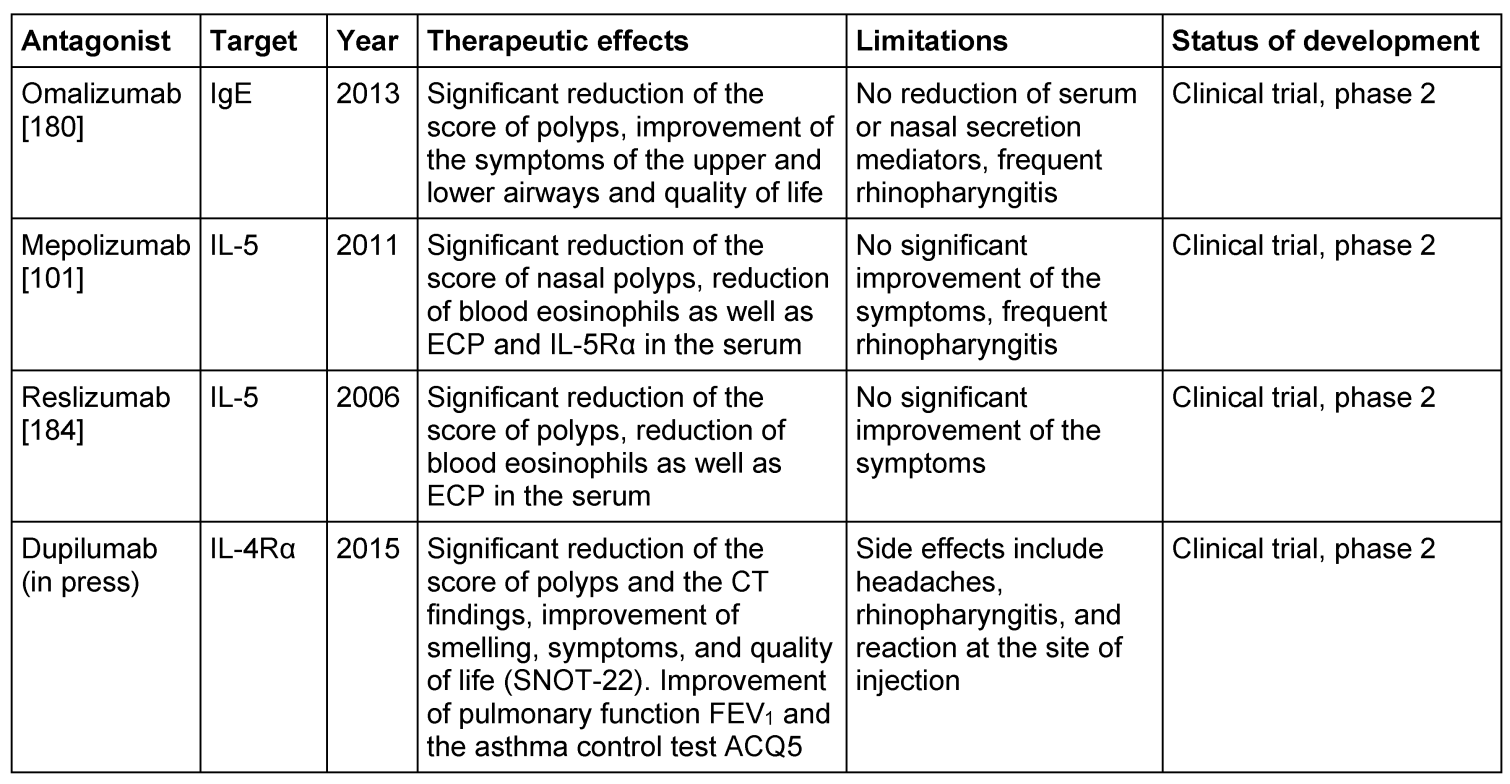
Biologics and other innovative therapeutic concepts for the treatment of CRSwNP
